# From sugar binders to diabetes fighters: the lectin saga of antihyperglycemic activity through systematic review and meta-analysis

**DOI:** 10.3389/fphar.2024.1382876

**Published:** 2024-09-11

**Authors:** Akshaya Simha N., Shashank M. Patil, Jayanthi M. K., Chaitra N., Ling Shing Wong, Jureerat Kijsomporn, Ranjith Raj, Ramith Ramu

**Affiliations:** ^1^ Department of Biotechnology and Bioinformatics, JSS Academy of Higher Education and Research, Mysuru, Karnataka, India; ^2^ Department of Pharmacology, JSS Medical College, JSS Academy of Higher Education and Research, Mysuru, Karnataka, India; ^3^Division of Medical Statistics*,* School of Life Sciences*,* JSS Academy of Higher Education and Research, Mysuru, Karnataka, India; ^4^ Faculty of Health and Life Sciences, INTI International University, Nilai, Malaysia; ^5^ Nursing School, Metharath University, Bangtoey, Pathum Thani, Thailand

**Keywords:** lectins, diabetes mellitus, antihyperglycemic, hormonal effects, carbohydrate-digesting enzymes, oxidative stress, insulin production process, toxicity

## Abstract

**Introduction:**

Lectins are carbohydrate-binding proteins that are extremely selective for sugar groups in the other molecules. As a result, they perform a variety of roles in biological processes involving cell, carbohydrate, and protein recognition at the cellular and molecular levels. Because lectins can bind to carbohydrates, they may play a role in determining the rate of carbohydrate digestion. They also bind to some proteins involved in diabetes mellitus (DM) pathophysiology. The present review aims to summarize the efficiency of lectins from different sources as potential antihyperglycemic agents.

**Methods:**

The Preferred Reporting Items for Systematic Reviews and Meta-Analyses (PRISMA) guidelines were employed for the drafting. In this regard, published scientific articles on the effects of different lectins on blood glucose (BG), glucose tolerance, hormonal effects, carbohydrate-digesting enzymes, oxidative stress, and insulin production process were collected from reputed journals using electronic databases. Furthermore, the toxicity effects of lectins from different sources were collected. A specific keyword search was completed to collect numerous articles with unique experimental designs and significant results. This was followed by the selection of the requisite articles based on the criteria designed by the authors. Data extraction was based on the common research elements included in the articles.

**Results and Discussion:**

Of 13 identified studies, 11 studies were considered after double screening based on the inclusion criteria. All 11 pharmacological investigations were considered for review. Subsequent studies reflected on the pharmacological properties of lectins on the levels of BG, oxidative stress, β-cell proliferation, insulin resistance, inhibition of carbohydrate digesting enzymes, body weight, food and water intake, lipid profile, and other parameters. This review highlights lectins as potential anti-diabetic agents.

**Conclusion:**

However, due to limited research, systematic evaluation is recommended for their development and promotion as effective potential antihyperglycemic agents. The clinical efficacy and safety of lectins against diabetes mellitus must also be evaluated.

## 1 Introduction

Diabetes mellitus (DM) is a chronic metabolic disorder that results from insufficient insulin production or the body’s inability to utilize insulin. It is classified into type 1 (insulin-dependent) and type 2 (insulin-independent) diabetes. Type 1 is caused by the autoimmune destruction of pancreatic beta cells, while type 2 diabetes is associated with insulin resistance or reduced insulin secretion (Ramu et al., 2022). In addition, DM causes other health maladies known as diabetic complications. These complications include cardiovascular diseases, kidney diseases, neuropathy, and blindness. Chronic hyperglycemia and insulin resistance are the root causes of these DM-associated complications (Martiz et al., 2022). The other causes for the development of DM are age, ethnicity, family history, smoking (oxidative stress), obesity, and physical inactivity (Maradesha et al., 2022). DM can be treated with oral hypoglycemic agents, including biguanides, thiazolidinediones, sulfonylureas, meglitinides, α-glucosidase inhibitors, and di-peptidyl peptidase-4 (DPP-4) inhibitors, which have varied mechanisms of action (Sajal et al., 2022). However, these drugs cause side effects like anemia, keto-acidosis, gastrointestinal problems, and other complications. Some phytochemical-based drugs are in clinical trials and have not yet been approved. These phytochemicals are difficult to extract, and the development of antihyperglycemic medications from these compounds is tedious (Patil et al., 2022a).

**FIGURE 1:**
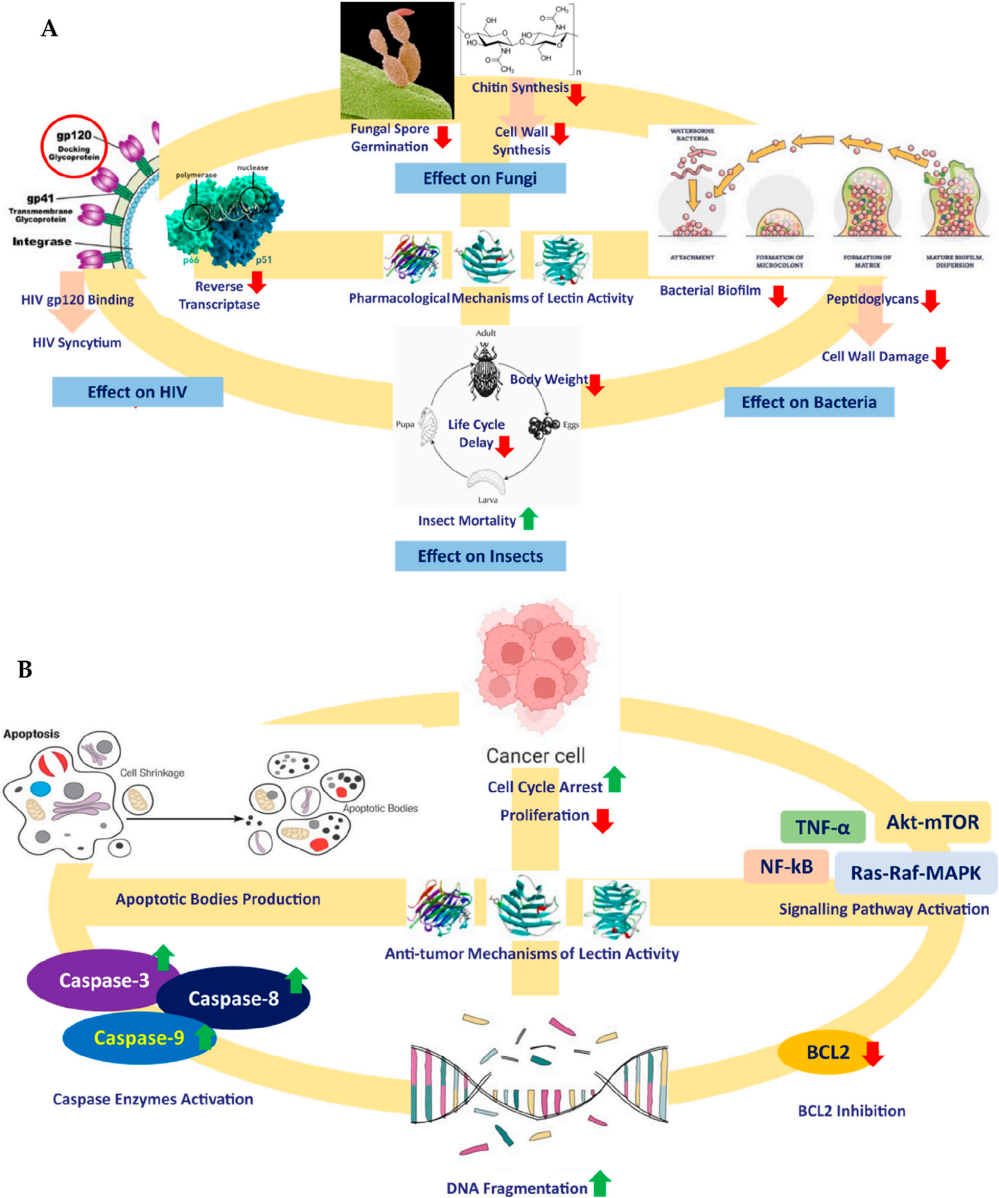
Mechanisms of different pharmacological **(A)** and antitumor **(B)** activities of lectins.

In this regard, biomolecules from living sources could play an important role in the modulation of glucose metabolism. Because of their origin from living sources, they are expected to cause minimal immunogenic response upon administration (Ramu et al., 2017; Kumar et al., 2021). These chiefly include carbohydrates, lipids, glycosides, proteins, peptides, and steroids. Among these, the anti-diabetic potential of carbohydrate-binding proteins or glycoproteins called lectins is important. The word “lectin” is a derivative of the Latin word “legere,” meaning “to select.” Lectins were first discovered by Peter Hermann Stillmark in 1888. They are also referred to as “hemagglutinins” when the bound sugar is unidentified. Lectins or hemagglutinins could be a protein or glycoprotein, consisting of a minimum single domain with no property of catalysis that displays reversible binding to definite sugar or oligosaccharides. Available literature depicts the other applications of lectins like antifungal, insecticidal (Fitches et al., 2008; Kaur et al., 2009; Jaber et al., 2007), antibacterial (Marques et al., 2018; Siritapetawee et al., 2018; Braga et al., 2015; Ramos et al., 2014), antifungal category (Ngai and Ng, 2007; Ramos et al., 2014), antiviral (Wang et al., 2006; Zhao et al., 2009) and anti-tumor activities (Cheung et al., 2009; Fang et al., 2010; Lam and Ng, 2010; Wu and Bao, 2013; Wang et al., 2008; Bhutia et al., 2008; Li et al., 2008) (Figure 1).

Over time, several researchers have focused on several aspects of the antihyperglycemic properties of lectins isolated from different plants. The literature survey in our study depicts their involvement in reducing blood sugar levels (Kavalali et al., 2002), serum glucose levels, and liver glycogen content (Sawant et al., 2017) in Wistar rats. When targeting hyperglycemia, both α-glucosidase and α-amylase activities were reduced by the intake of lectin. It also reduced the sucrase levels *in vitro*. Lectins were even found to improve the condition of the islets of Langerhans to improve insulin production (Govindappa et al., 2015; Wang et al., 2012; Alves et al., 2020). Another study proved that lectins have insulin-mimicking properties (Sawant et al., 2017). Kidney function markers like urea and creatinine levels were found to be reduced in male albino Wistar rats upon treatment with lectins (Govindappa et al., 2015). In these few incidences, diabetic symptoms were reduced. In addition, obesity/hyperlipidemic factors like serum cholesterol and triglycerides were also reduced in animal models upon treatment with lectins (Sawant et al., 2017). Therefore, lectins could be treated as potential biomolecules for treating diabetes mellitus.

With the increasing rate of adverse effects caused by chemical antihyperglycemics and the tedious procedure of phytochemical-based drug discovery, studies focusing on safer and more reliable alternatives are encouraged. In this regard, our primary objective is to systematically gather, organize, and present data on the antihyperglycemic activity of lectins using various research models and approaches. This comprehensive approach will contribute to the advancement of research in this field. Disseminating the findings from this systematic review and meta-analysis could provide valuable insights to researchers, thereby enhancing the quality of research.

This review is the first of its kind to report on the anti-diabetic properties of lectins, focusing on both *in vivo* and clinical trials. In this review, we emphasize the effects of lectins on BG, glucose tolerance, hormonal effects, carbohydrate-digesting enzymes, oxidative stress, and insulin production process, ultimately targeting DM. We also review and comment on toxicological studies of the reported antihyperglycemic lectins. This review highlights the overall efficacy of lectins as potential antihyperglycemic agents that could compete with conventional therapeutics.

## 2 Materials and methods

**FIGURE 2:**
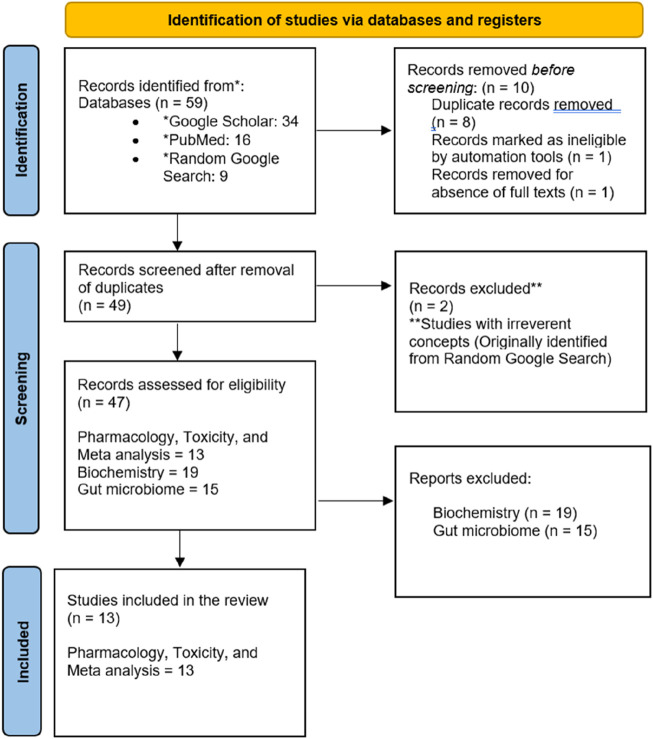
PRISMA flowchart depicting the literature management.

The drafting of this review followed the recommendations and guidelines of Preferred Reporting Items for Systematic Reviews and Meta-Analysis (PRISMA). Procedures were followed by the authors before framing the article (Figure 2). Following this, inconsistencies were solved by the second author, and the third author reviewed the framed article. The materials and methods employed in this systematic review and meta-analysis were derived from the authors’ prior research endeavors (Patil et al., 2021a; Patil et al., 2021b; Patil et al., 2022b).

### 2.1 Databases, software, e-sources, and keywords search

An extensive and comprehensive literature search was executed to collect available information surrounding the antihyperglycemic potential of lectins. The literature survey was completed between 1 January and 15 February 2024. The drafting and serial revision were completed between 17 February and 28 February 2024. The survey was conducted to collect information about the anti-diabetic properties of lectins using various electronic databases, including Scopus, EMBASE, Google Scholar, PubMed, Springer Link, Wiley-Blackwell, and Web of Science. Hence, the literature survey ended with many studies published in peer-reviewed journals, patents, and conference proceedings. The orderly arrangement and categorization of literature were carried out with the support of Mendeley. Keywords such as “lectins and anti-diabetic property,” anti-diabetic lectins, “lectins and anti-hyperglycemic,” “lectins with hypoglycemic property,” “plant lectins and anti-diabetic,” “animal lectins and anti-hyperglycemic,” “toxicology of lectins” etc. were used to search for the articles.

### 2.2 Inclusion and exclusion criteria

A thorough literature search identified several relevant research papers, book chapters, brief communications, and Ph.D. theses. The following selection criteria were used: research that was exclusively published in English, had relevant study titles, abstracts, and keywords, and dealt with effects of lectins on BG, glucose tolerance, hormonal effects, carbohydrate-digesting enzymes, oxidative stress, insulin production process, and lectin toxicity analysis. The articles were completely screened by two authors using the selection criteria. The enormous body of literature was sorted into various areas based on the titles and abstracts that were accessible, and the duplicates and studies that contained irrelevant data were deleted. Studies with original methodologies that covered all relevant study topics were retained after a second screening. A few articles with limited information were removed. A few additional research publications were excluded, while the review was written to retain only those with original methodologies and noteworthy findings. Due to a lack of pertinent information in other sources, several articles were retained despite their unimportant results and outdated methods. The screening was completed as the authors reached a consensus about the final amount of literature.

### 2.3 Data extraction and analysis

All the selected manuscripts were analyzed as per the following criteria: year of publication, type of pharmacological effect, study type (*in vivo* or *in vitro*), animal models used, source of lectin used, dosage given, controls used, results obtained, and the underlying mechanism underlying the respective pharmacological activity. To assess the pharmacological activity, the authors paid close attention to the type of lectins employed. Studies were also conducted to determine what toxicity data were available. A list of the studies was then created, along with detailed annotations. Furthermore, all the literature gathered was divided into categories based on its pharmacological effect on diabetes mellitus. All the articles from the categorized groups were summarized to give a cumulative representation that could help the reader understand the pharmacological effects induced by lectins in different study models.

## 3 Results and discussion

**FIGURE 3:**
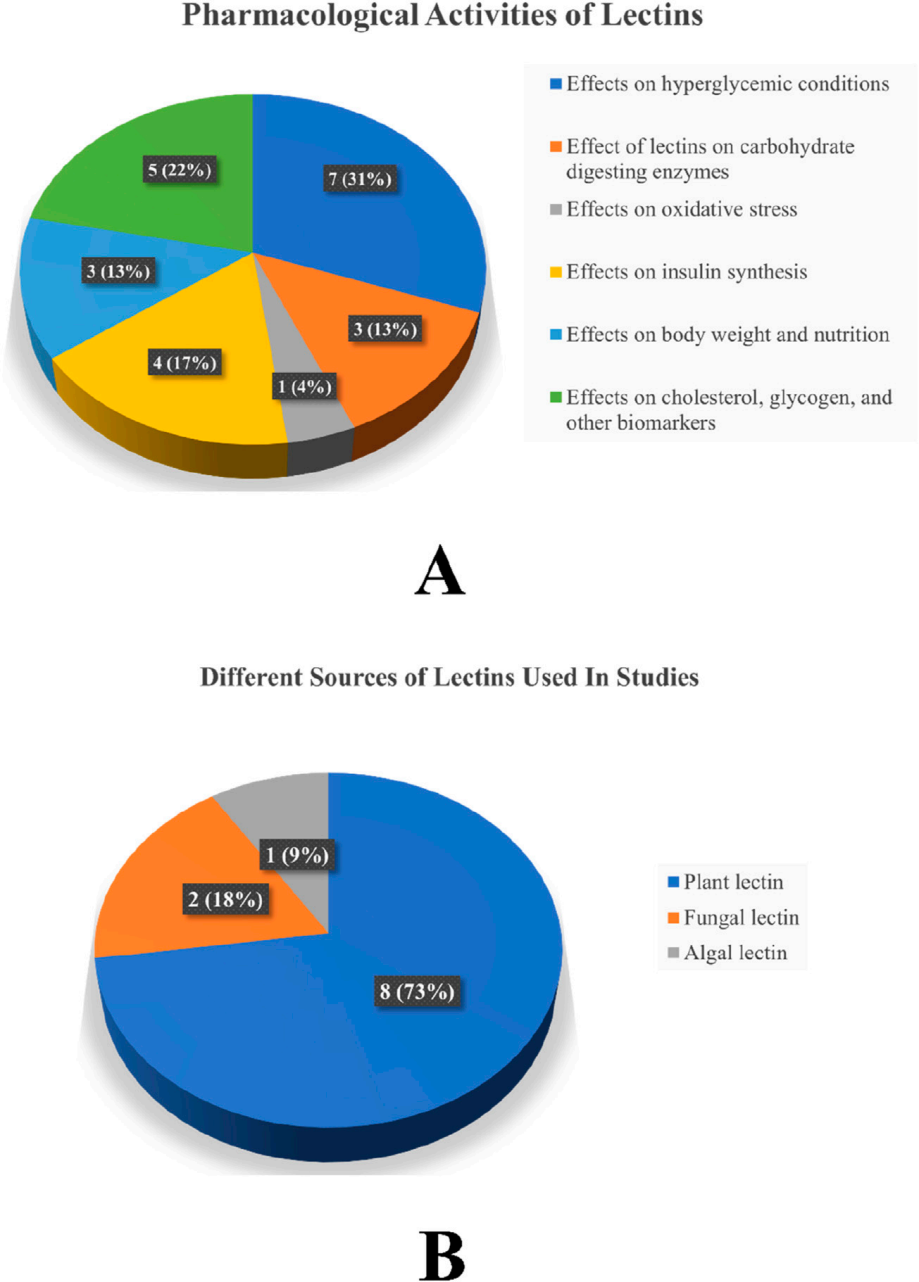
**(A)** Different pharmacological activities of lectins in antihyperglycemic activity and **(B)** different sources of lectins used in studies. *Note*: The values represent both the number of studies and the percentage.

Thirteen articles were retained after removing duplicates. Of the 13 articles, two were excluded: one for the availability of abstract only and the other because it addressed information other than anti-diabetic properties of lectin. After screening, a total of 11 articles with required pharmacological evaluations were considered for the preparation of the review article. Most of the studies were completed between 2002 and 2021. Of the 11 articles, 32% described the effect of lectins on BG, serum cholesterol, glycogen, and other biomarkers, 16% evaluated the effect of lectins on glucose tolerance, 10% of the articles studied the effect of lectins on body weight, food and fluid intake, and hormone release. Furthermore, 16% dealt with the effect of lectins on carbohydrate-digesting enzymes, 21% dealt with the effect of lectins on oxidative stress, and only 5% of the articles described the effect of lectins on the number of islet cells, β-cell proliferation, gene regulation, and insulin resistance. The results of all pharmacological evaluations of lectins are summarized in Table 1. A pie chart depicts the different sources of lectins and their pharmacological actions in antihyperglycemic activity (Figure 3). Approximately 73% (8) of the research articles did not report toxicity tests, and the remaining 27% (3) did. Approximately 55% of the research work related to lectins and anti-diabetic properties was carried out in Asia; Europe contributed to approximately 18% of the work, and 27% of the work was carried out in South America. Approximately 55% of the research work mentioned the sample size, whereas 45% of the studies did not. The authors categorized the literature based on the reported antihyperglycemic activity. Seven selections were made based on the type of pharmacological activity depicted in the collected articles, namely, i) Effects on hyperglycemic conditions, ii) Effects on carbohydrate-digesting enzymes, iii) Effects on oxidative stress, iv) Effects on insulin synthesis, v) Effects on body weight and nutrition, vi) Effects on cholesterol, glycogen, and other biomarkers, and vii) Effects on gut microbiome and diabetes mellitus. The studies that reported the toxicity effects of the lectins were also considered in a separate section. The summary table was prepared after categorization. The reviewing and addition of the authors’ perspectives toward these sections is the core of this systematic review. The meta-analysis was conducted on six (Kavalali et al., 2002; Govindappa et al., 2015; Sawant et al., 2017; Alves et al., 2020; Vera-Nuñez et al., 2021; Rocha et al., 2013) of the eleven articles that involved *in vivo* experiments. The articles that mentioned sample size in their methodology were considered for the meta-analysis.

**TABLE 1:** Summary of different pharmacological activities of lectins isolated from different sources.

Type of study	Models	Type of lectin	Source of lectin	Mode of administration	Doses	Controls	Possible mechanism	Results	Reference
Effects on hyperglycemic conditions									
*In vitro* and *in vivo*	Male Wistar rats	Plant lectin *Urtica pilulifera* (UPSL)	Seeds	Intra-peritoneal	100 mg/kg (lectin) 10 mg/kg (glipizide)	Saline and glipizide	Competitive inhibition of STZ by UPSL	BG levels (initial and 4th week) BG in groups III and IV decreased at the end of the 4th week. The diabetic group had a BG level of 412.42 ± 111.65 mg/dL, whereas the lectin-treated group showed a reduction in BG level (296 ± 89.34), and the group treated with the standard drug had 273.37 ± 55.75	Kavalali et al. (2002)
*In vivo*	Female Wistar-rats	Plant lectin *Abrus precatorius* (ABA)	Seeds	Oral	150 mg/kg 200 mg/kg	Saline, sodium carpometacarpal (CMC), and pioglitazone	Insulin-mimicking property	Serum glucose levels (mg/dL) on Day 0, day 7, and day 14 Serum glucose levels increased in the diabetic control group by 137% and 140% on the 7th and 14th day, respectively, compared to the normal group. However, in the treated group, serum glucose levels were decreased by 15% and 40% and 13% and 43% for the ABA 150 and ABA 200 groups on the 7th and 14th day, respectively, compared to the diabetic group The liver glycogen level was reduced by 21% and 26% due to lectin treatment at leptin doses of 150 mg/kg and 200 mg/kg, respectively, whereas the pioglitazone-treated group experienced a 15% reduction of the glycogen level	Sawant et al. (2017)
*In vivo*	Female albino Swiss mice	Plant lectin *Crataeva tapia* (CrataBL)	Bark	Intra-peritoneal	10 mg/kg and 20 mg/kg	Saline and insulin	Not mentioned	Effect of CrataBL on fasting glucose CrataBL proved to be an effective hypoglycemic agent and exhibited excellent antihyperglycemic activity after 10 days of treatment. A 20 mg/kg dose of the lectin had a better (56%) decrease in glucose level than the 10 mg/kg dose, which was only 15%. Similarly, the standard drug insulin exhibited a 64% reduction with no significant difference	Rocha et al. (2013)
*In vivo*	Wistar rats	Plant lectin *Vigna radiata*	Seed	Oral	150 mg/kg 250 mg/kg 300 mg/kg	Glucose Glibenclamide	Insulinomimmetic	Serum glucose level was decreased by a lectin isolated from *Vigna radiata* bound with zinc (zinc-lectin complex) at a lower dose than individual lectins administered through oral and subcutaneous routes	Gadgoli et al. (2015)
*In vitro* and *in vivo*	Glucose diffusion method and male albino Wistar rat	Endophytic fungal lectin *Alternaria* species	Leaf	Oral	400 mg/kg	Saline, glucose (to prevent hypoglycemia), and glibenclamide	Not mentioned	Effect of lectin on serum glucose levels (Days 1, 7, and 14) At 400 mg/kg, glucose was decreased by 60.76% on the 14th day, that is, from 330.40 ± 7.44 to 129.62 ± 09.10 mg/dL. Effect of lectin on kidney function markers After 14 days of treatment at a dose of 40 mg/kg, significant reductions in urea (15.9%) and creatinine (0.08%) levels were observed. After the lectin treatment, serum aspartate transaminase (AST) and alanine transaminase (ALT) levels were reduced by 26% and 5%, respectively. Effect of lectins on serum cholesterol and triglycerides After 14 days of treatment, the lectin-treated group showed serum cholesterol and triglyceride values of 90.04 ± 0.92 and 98.51 ± 2.02 mg/dL, respectively. The diabetic-induced group showed serum cholesterol and triglyceride levels of 153.26 ± 1.45 and 176.64 ± 2.62 mg/dL, respectively. The lectin-treated diabetic group exhibited reduced serum cholesterol and triglyceride levels of 103.54 ± 2.13 and 124.68 ± 2.49 mg/dL. The standard glibenclamide group exhibited reduced serum cholesterol and triglyceride levels of 98.44 ± 1.28 & 110.44 ± 2.02 mg/dL, respectively. Although the serum cholesterol level after lectin treatment is slightly higher than the standard, lectin effectively decreases serum cholesterol levels	Govindappa et al. (2015)
*In vivo*	Male mice (CD-1)	Plant lectin *Lupinus albus*	Seeds	Oral	60 mg/kg	Water, glucose, and metformin	Binding of lectin to HepG2 cell glucose receptors, insulin receptors, and human insulin	Effect of lectin on glucose level After 120 min, the BG level reduced to the basal level	Grácio et al. (2021)
*In vivo*	C57BL/6J mice	Fungal lectin *Agaricus bisporous* (ABL)	Not mentioned	Not mentioned	10 mg/kg (ABL) D-glucose (1 g/kg)	Saline	Β-cell proliferation via regulation of cell cycle proteins and upregulation of genes involved in β-cell replication	By the end of the 120-min testing period, glucose concentration remained >350 mg/dL in the PPx control mice after 7 days of recovery. In contrast, the glucose concentration reached ≤300 mg/dL by the end of the 120 min testing period in ABL-treated mice after 7 days of therapy. Glucose tolerance tests performed on day 14 revealed significant differences between the PPx control mice and the ABL-treated mice by the end of the 120-min testing period	Wang et al. (2012)
Effects on carbohydrate-digesting enzymes									
*In vitro*	Not defined	Plant lectin *Archidendron jiringa*	Seeds	α-Glucosidase	pNPG (0.025–0.2 mM) 0 mg/mL, 0.05 mg/mL, and 0.075 mg/mL of lectin	Phosphate buffer	Non-competitive inhibition of alpha-glucosidase	Both K_m_ and V_max_ decreased with increased concentration; hence, GI acted as a non-competitive inhibitor. IC_50_ was 0.031 ± 0.02 mg/mL K_i_ was1.887 μg/mL	Virounbounyapat (2012)
*In vitro*	Not mentioned	Endophytic fungal lectin *Alternaria* species of *Viscum album*	leaf	α-Amylase α-Glucosidase sucrase	FRB–1.5% Sample emulsion–10.50 µL	Not mentioned	Inhibition of three digestive enzymes	α-Amylase inhibitory activity was studied using the α-amylase star model with an inhibition of 85.26% ± 1.25%. α-Glucosidase was significantly inhibited by lectin, and it was 93.41% ± 1.27%. Sucrase inhibition was 81.61% ± 1.05%	Govindappa et al. (2015)
*In vitro*	Not mentioned	Plant lectin *Sterculia monosperma*	Vent seeds	α-Glucosidase	10 µL lectin–0 mg/mL, 0.05 mg/mL, and 0.075 mg/mL 10 µL 1 mM pNPG–0.025–0.2 mM	Not mentioned	Non-competitive inhibition of α-glucosidase	Both Km and Vmax decreased with increased concentration; hence, GI acted as a non-competitive inhibitor with a Ki value of 1.39 μg/mL	Karnchanatat and Sangvanich (2014)
Effects on oxidative stress									
*In vitro* and *in vivo*	Male Wistar rats	Algal lectin *Bryothamnion seaforthii*	Not mentioned	Oral	BSL (600 μg/kg, 300 μg/kg, and 150 μg/kg	150 mM NaCl Metformin (600 μg/kg) STZ (60 mg/kg)	Activation of antioxidant enzymes	1) Superoxide dismutase Blood from days 30, 60, 90, and 120 showed significant increases in the enzymatic activity in the BSL-treated group for all concentrations compared to the negative control and metformin groups. 2) Catalase The enzymatic activity of CAT was found to not change significantly among the groups. 3) Glutathione peroxidase An insignificant increase was noted in the enzyme activity across all BSL-treated groups from the blood collected at 30, 60, 90, and 120 days. After 90 and 120 days, the BSL group (600 μg/kg) had a significant difference compared to the other BSL groups (150 μg/kg and 300 μg/kg)	Alves et al. (2020)
Effects on insulin synthesis									
*In vivo* histopathological study	Male Wistar rats	Plant lectin *Urtica pilulifera*	Seeds	Intra-peritoneal	100 mg/kg (lectin) 10 mg/kg (glipizide)	Saline Glipizide	Competitive inhibition of STZ by UPSL	The number of islets and islet cells was found to be lower than in the normal group, and the average of the diabetic control was statistically significant. The size of the islets was small in group IV	Kavalali et al. (2002)
*In vivo* immunohistological study	C57BL/6J mice	Fungal lectin’ *Agaricus bisporus*	Not mentioned	Not mentioned	10 mg/kg (ABL)	Saline	Β-cell proliferation via regulation of cell cycle proteins and upregulation of genes involved in β-cell replication	Effect of ABL on β-cell proliferation The pancreas section stained for BrdU and insulin showed a remarkable increase in the number of BrdU-positive cells treated by ABL (*Agaricus bisporous* lectin). A significant increase in beta-cell proliferation was observed with an increase of 2.7 folds. In ABL-treated mice, small islets had greater proliferation rates than the larger ones. Analysis of β-cell mass ABL-treated mice had elevated serum insulin concentrations compared to PPx control mice. Absolute β-cell mass was significantly elevated by ABL treatment. Analysis of cyclin D-Cdk4 complex A remarkable increase in cDK4 mRNA and protein concentrations was observed compared to the pramipexole (PPx) control by RT-PCR. mRNA and protein concentrations of cyclin D1 and cyclin D2 were also increased. Cyclin D2 upregulated in ABL-treated mice. Analysis of cyclin-dependent kinase 4 (Cdk4) activity In ABL-treated mice, phosphorylation of rGST-Rb was significantly higher in islet lysates than in PPx control mice, indicating that Cdk4 was activated. Analysis of the abundance of β-cell-specific genes. Significant enhancement in insulin, GLUT-2, and glucokinase mRNA levels by ABL treatment	Wang et al. (2012)
*In vitro* and *in-vivo*	Male Wistar rats	Algal lectin *Bryothamnion seaforthii*	Not mentioned	Oral	BSL (600 μg/kg, 300 μg/kg and 150 μg/kg	150 mM NaCl Metformin (600 μg/kg) STZ (60 mg/kg)	Decreases insulin resistance, enhances modifications in lipid metabolism (decreases lipidic blood flow), and reduction of free radicals	HOMA method to evaluate insulin resistance On day zero, all groups were within the same value of insulin resistance of 5% significance values. At 30 days, 60 days, 90 days, and 120 days, a significant decrease in IR was noted in the BSL and metformin-treated groups compared to the negative control. HOMA-β method to evaluate the functional capacity of β-cells In the beginning, all groups experienced insulin hypersecretion. However, with BSL treatment at three different doses, there was a decrease compared to the normal group (increased hypersecretion), which remained constant for the metformin group	Alves et al. (2020)
*In vivo*	Male C57BL/6 mice	Plant lectin: *Moringa oleifera*	Seeds	Not mentioned	WSMol (5 mg/kg)	HFD (45%) + STZ (40 mg/kg) 0.2 mL Saline 0.5 IU/kg of insulin	Insulin resistance	Three weeks of WSMol treatment was sufficient to decrease the insulin resistance in the T2DM group and the control non-diabetic group.	Vera-Nuñez et al. (2021)
Effects on body weight and nutrition									
*In vitro* and *in vivo*	Male Wistar rats	Plant lectin *Urtica pilulifera* (UPSL)	Seeds	Intra-peritoneal	100 mg/kg	Saline and glipizide	Competitive inhibition of STZ by UPSL	Body weight in grams (initial and 4th week) Similarities in initial body weight were observed in normal and diabetic groups. The diabetic groups had lower body weight than the normal group, but group III showed better results than the other diabetic groups, that is, groups II and IV. Effect of lectin on food intake Food intake was high in the diabetic groups compared to the normal group. Effect of lectin on fluid intake Fluid intake was also high in the diabetic groups compared to the normal group	Kavalali et al. (2002)
*In vivo*	Male albino Wistar rats	Endophytic fungal lectin *Alternaria* species	Leaf	Oral	400 mg/kg	Saline, glucose (to prevent hypoglycemia), and glibenclamide	Not mentioned	Effect of lectin on body weight Body weight was increased by 8.50%, which was significant	Govindappa et al. (2015)
*In vivo*	Female Wistar rats	Plant lectin *Abrus precatorius* (ABA)	Seeds	Oral	150 mg/kg 200 mg/kg	PBS, sodium CMC, and pioglitazone	Insulin-mimicking property	Effect on food intake in ABA-treated diabetic animals On day 14, food intake was significantly reduced to 69% compared to the normal group. In contrast, food intake of the ABA-150, ABA-200, and standard groups was enhanced by 103%, 162%, and 182%, respectively. Effect on body weight in ABA-treated diabetic animals Body weight was increased by 17% and 31% on the 7th and 14th days, respectively, after treatment with ABA-150. Similarly, body weight increased by 20% and 32% on the 7th and 14th days, respectively, after the treatment with ABA-200	Sawant et al. (2017)
Effects on cholesterol, glycogen, and other biomarkers									
*In vivo*	Female Wistar rats	Plant lectin *Abrus precatorius*	Seeds	Oral	150 mg/kg 200 mg/kg	Saline, sodium CMC suspension, and pioglitazone	Insulin-mimicking property	Serum cholesterol level (mg/dL) – Day 0, day 7, and day 14 Serum cholesterol levels increased by 75% and 79% on the 7th and 14th days, respectively, compared to the normal control group. SCL decreased by 16% and 33% on the 7th and 14th days, respectively, in the ABA-150-treated group compared to the diabetic group. SCL decreased by 19% and 36% on the 7th and 14th days, respectively, in the ABA-200-treated group compared to the diabetic group Liver glycogen content A remarkable decrease of 75% in liver glycogen levels was noted in the diabetic control group compared to the normal control group. The ABA-150, ABA-200, and PIO-treated groups exhibited a reduction in glycogen by 21%, 26%, and 15%, respectively, compared to the normal group. Still, there was an increase in glycogen level, almost reaching the normal level, in the PIO case	Sawant et al. (2017)
*In vivo*	Wistar rats	Plant lectin *Vigna radiata*	Seed	Oral	150 mg/kg 250 mg/kg 300 mg/kg	Glucose Glibenclamide	Insulinomimmetic	Individual lectin and lectin complexed with zinc reduced the serum cholesterol and triglyceride levels; however, lectin complexed with Zn exhibited a higher effect at lower doses than lectins. Lectin and lectin complexed with zinc re-established liver glycogen levels	Gadgoli et al. (2015)
*In vivo* immunohistological study	C57BL/6J mice	Fungal lectin’ *Agaricus bisporus*	Not mentioned	Not mentioned	10 mg/kg (ABL)	Saline	Β-cell proliferation via regulation of cell cycle proteins and upregulation of genes involved in β-cell replication	Effect of ABL on β-cell proliferation Pancreas sections stained for BrdU and insulin showed a remarkable increase in the number of BrdU-positive cells treated by ABL (*Agaricus bisporous* lectin). A significant increase in beta-cell proliferation was observed with an increase of 2.7 folds. In ABL-treated mice, small islets had greater proliferation rates than the larger ones. Analysis of β-cell mass ABL-treated mice had elevated serum insulin concentrations compared to PPx control mice. Absolute β-cell mass was significantly elevated by ABL treatment. Analysis of cyclin D-Cdk4 complex A remarkable increase in cDK4 mRNA and protein concentrations was observed compared to the pramipexole (PPx) control by RT-PCR. mRNA and protein concentrations of cyclin D1 and cyclin D2 were also increased, respectively. Cyclin D2 was upregulated in ABL-treated mice. Analysis of cyclin-dependent kinase 4 (Cdk4) activity In ABL-treated mice, phosphorylation of rGST-Rb was significantly higher in islet lysates than in PPx control mice, indicating that Cdk4 was activated. Analysis of the abundance of β-cell-specific genes. A significant enhancement in insulin, GLUT-2, and glucokinase mRNA levels after ABL treatment was noted	Wang et al. (2012)
*In vitro* and *in vivo*	Male Wistar rats	Algal lectin *Bryothamnion seaforthii*	Not mentioned	Oral	BSL (600 μg/kg, 300 μg/kg, and 150 μg/kg)	150 mM NaCl Metformin (600 μg/kg) STZ (60 mg/kg)	Decreases insulin resistance, enhances modifications in lipid metabolism (decreases lipidic blood flow), and reduces free radicals	HOMA method to evaluate insulin resistance On day 0, all groups were within the value of insulin resistance of 5% significance values. At 30 days, 60 days, 90 days, and 120 days, a significant decrease in IR in the BSL and metformin-treated groups compared to the negative control was noted. HOMA-β method to evaluate the functional capacity of β-cells In the beginning, all groups experienced insulin hypersecretion. However, with BSL treatment at three different doses, there was a decrease compared to the normal group (increased hypersecretion), which remained constant for the metformin group	Alves et al. (2020)
*In vivo*	Male C57BL/6 mice	Plant lectin: *Moringa oleifera*	Seeds	Not mentioned	WSMol (5 mg/kg)	HFD (45%) + STZ (40 mg/kg) 0.2 mL saline 0.5 IU/kg of insulin	Insulin resistance	Three weeks of WSMol treatment was sufficient to decrease the insulin resistance in the T2DM group as well as in the control non-diabetic group	Vera-Nuñez et al. (2021)

## 4 Biochemistry of lectins

Lectins differ from other proteins, such as immunoglobulins that agglutinate cells, in terms of their amino acid composition, involvement of metals, three-dimensional structure, and molecular weight. Lectins are a class of proteins that are ubiquitous in animals, plants, and bacteria. These proteins can establish reversible bonds with cells by interacting with carbohydrates or glycoproteins present in fluids or on cell membranes. Lectins exhibit specificity toward monosaccharides or oligosaccharides, and their activity can be inhibited by oligosaccharides, glycoproteins, or polysaccharides (Wälti et al., 2008; Thakur et al., 2007).

Due to the presence of conserved amino acid sequences present in the binding site, the selectivity toward carbohydrates can be observed among the lectin families (Peumans and Damme, 1998). Due to high similarity in their residues, including those involved in binding to monosaccharides, these proteins can have metal ions and interactions coordinated by water molecules and carbohydrates. They can present 2 to 12 interaction sites, depending on the molecule’s nature and oligomerization state. Structural differences occur in lectins from the primary structure to the last degree of molecular organization, including amino acid sequence, number of subunits, and polypeptide nature. Interactions between subunits play a dominant role in the stability of these proteins. Specificities and affinities of the associated sites are achieved mainly by hydrogen bridges, including van der Waals and hydrophobic interactions with aromatic amino acids close to hydrophobic monosaccharide residues (Sharon, 1993; Sharon and Lis, 2002; Balzarini, 2006; Mitra et al., 2002). Hydrogen bonds, along with van der Waals’ forces and hydrophobic interactions with aromatic amino acid residues near monosaccharide hydrophobic portions, contribute to the specificities and affinities of the associated sites, ensuring the stability and specificity of the formed complexes (Sharon, 1993). Molecular dynamics simulations reveal that lectins are structurally flexible and adaptable to experimental differences in analyzing adaptable molecules. The presence of ions in the environment can determine the interaction between lectins and carbohydrates, and the concentration of ions may lead to the formation of micelles in the hydrophobic sites of lectins. The complexity of molecule binding is stabilized by hydrogen bridges, and specific and favorable electrostatic interactions between amino acid residues of the lectin and ligand monomers facilitate binding. These interactions contribute to the strength and stability of the molecular complex (Konidal and Niemeyer, 2007).

### 4.1 Plant lectins

**FIGURE 4:**
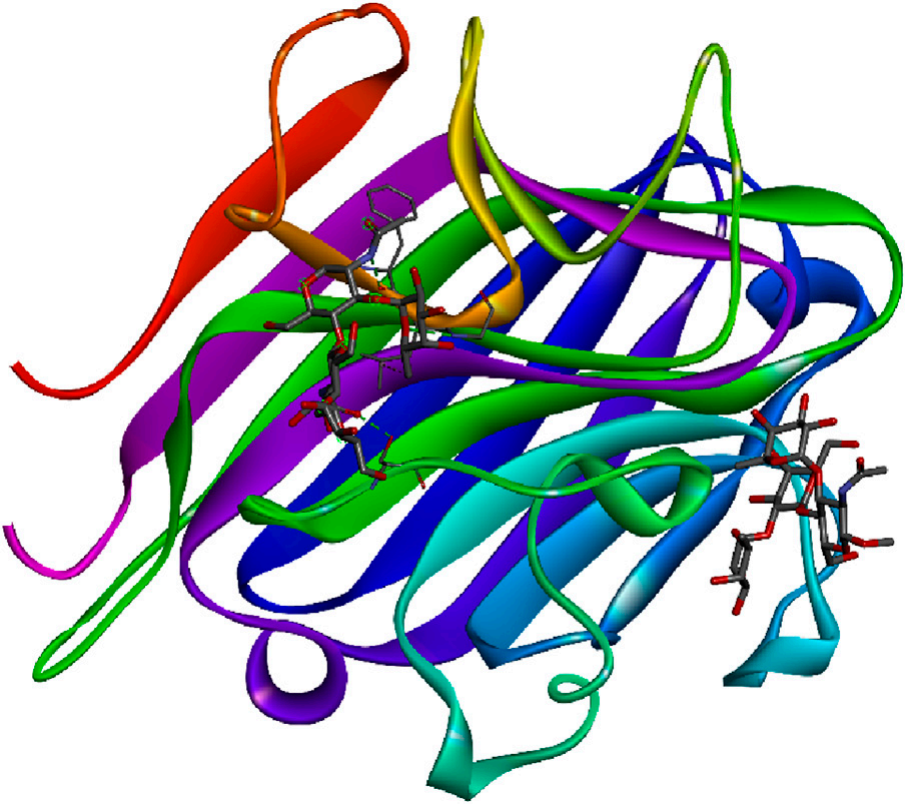
Plant lectin isolated from *Griffonia simplicifolia* bound with N-acetyl glucosamine, fucose, and galactose; structure obtained from UniProt (UniProt ID: P24146); image visualized using Biovia Discovery Studio 2021.

Based on structural and evolutionary similarities, plant lectins are divided into seven families: lectins of phloem from Cucurbitaceae, chitin-binding lectins, ribosome-inactivating protein type 2, mannose-binding lectins of monocots, jacalin related lectins, and the family of amarantins. The last three lectins are structurally similar with β structures only. Based on their overall structure, plant lectins are categorized into four distinct classes (Peumans and van Damme, 1995). Merolectins are small monovalent proteins with one carbohydrate-binding domain, incapable of precipitating or agglutinating cells. Hololectins have multiple carbohydrate-binding domains, capable of agglutinating cells or precipitating glycoconjugates. Chimerolectins, resulting from protein fusion, have one carbohydrate-binding domain and another independent domain. Superlectins have two or more distinct domains. Plant lectins can also be classified based on their interactions with various sugars, including mannose/glucose, galactose, mannose, fucose, and sialic acid (Wong et al., 2006; Bari et al., 2013). Figure 4 represents the 3D image of a plant lectin isolated from *Griffonia simplicifolia.*

### 4.2 Animal lectins

Invertebrate creatures, such as protozoa, insects, mollusks, crustaceans, sea cucumbers, polychaetes, and sea sponges, contain lectins in their hemolymph and coelomic fluid. Lectins have been identified and characterized in vertebrates such as fish, snakes, and mammals. Lectins from numerous tissues and cells in humans, including the lungs, serum, and dendrites, have been thoroughly studied (Santos et al., 2014).

Based on the location, animal lectins are divided into integral lectins of membranes and soluble lectins present in intra and intercellular fluids. Integral lectins are structural components of membranes and differ in specificity to carbohydrates and their physical and chemical properties. Soluble lectins can move freely in the intra- and intercellular environments. Gabius (1997) classified animal lectins into five categories based on their structural characteristics: C-type lectins, I-type lectins, galectin, pentraxins, and P-type lectins.

C-type lectins are metal-activated lectins that are dependent on the presence of Ca^2+^ ions for carbohydrate binding. They exhibit distinct selectivity and possess conserved carbohydrate recognition domains. I-type lectins have a carbohydrate recognition domain similar to immunoglobulins, while S-type lectins are thiol-dependent and bind to β-galactosides only. Pentraxins are composed of many subunits, while P-type lectins have a similar but not well-defined carbohydrate recognition domain (CRD) and are selective to glycoproteins with mannose 6-phosphate. P-type lectins, which include cation-dependent mannose 6 phosphate receptor (CD-MPR) and cation-independent mannose 6 phosphate receptor (CI-MPR), feature a phosphate group. These lectins primarily facilitate the transport of newly synthesized soluble acid hydrolyses to the lysosome by binding to mannose 6-phosphate residues on the N-linked oligosaccharides of the hydrolyses. The discovery of mannose 6 phosphate receptors (MPRs) arose from studies on mucolipidosis II, where it was observed that fibroblasts from affected individuals could uptake lysosomal enzymes from healthy cells due to a recognition tag, later identified as MPRs.

CI-MPR, a dimeric protein weighing approximately 300 kDa, shares structural similarities with CD-MPR but differs in being cation-independent. Unlike CD-MPR, CI-MPR interacts with proteins carrying the MPR tag, IFG-II, and other hydrolases. It’s three N-terminal domains form a tri-lobed disk crucial for maintaining the structure of sugar binding site. CI-MPR exhibits a relatively uniform binding affinity toward glycans with one or two phosphomonoesters, unlike CD-MPR, which shows a preference for glycans with two phosphomonoesters. Additionally, CI-MPR binds to ligands at the cell surface, unlike CD-MPR. Although both CD-MPR and domain 3 of CI-MPR share conserved residues crucial for mannose-6-P binding, differences exist, such as the absence of Mn^2+^ in CI-MPR’s binding pocket, possibly contributing to its cation-independence. Rhamnose-binding lectins (RBLs) are an animal lectin family with specific sugar-binding and a molecular structure consisting of two to three homologous, tandemly repeated CRDs (Jimbo et al., 2007). CD-MPR, a homodimer weighing 46 kDa, relies on cations for its function. The folding of its homodimer structure depends on the three disulfide linkages formed by six cysteine residues in the extracellular region. Despite differences, the tertiary structures of CD-MPR and CI-MPR are similar, given the similarity in size and amino acid sequence among their 15 contiguous domains in the extracellular region. Notably, several domains of CI-MPR share the same fold as specific domains of CD-MPR.

**FIGURE 5:**
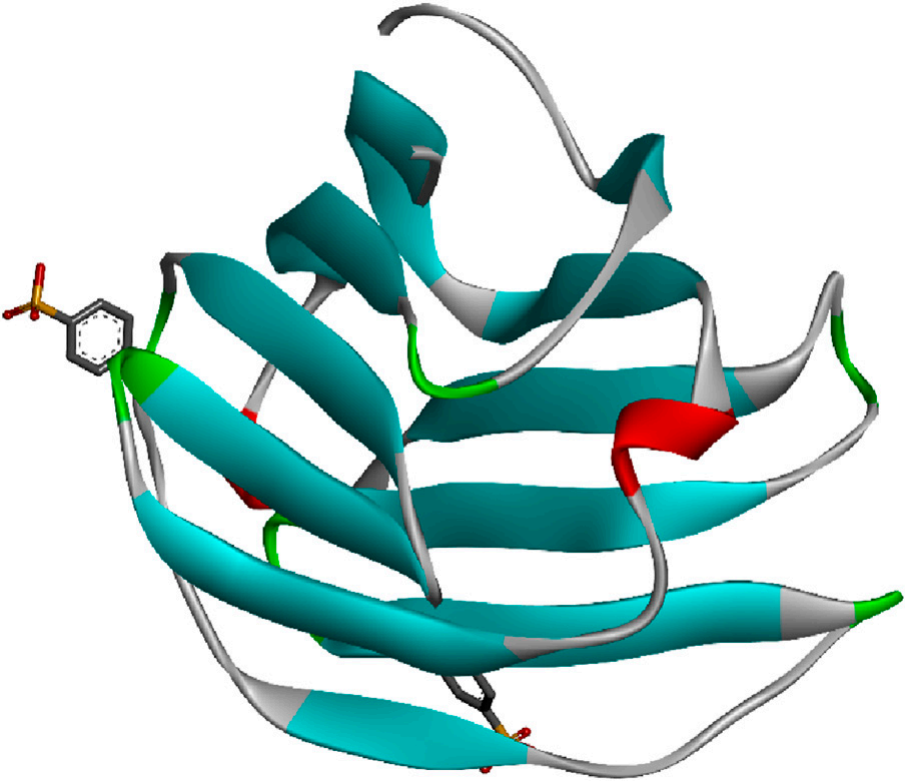
Animal lectin isolated from *Homo sapiens*; structure obtained from UniProt (UniProt ID: Q05315); image visualized using Biovia Discovery Studio 2021.

The monomeric structure of CD-MPR forms a flattened β-barrel comprised of two antiparallel β sheets, one containing four strands and the other five. In its dimeric form, CD-MPR exhibits two antiparallel β sheets, each containing five strands. Through mutagenesis studies, certain amino acids like E133, Y143, Q66, and R111 have been identified as crucial for mannose-6-P binding. The binding and unbinding mechanism of CD-MPR resembles the oxy-to-deoxy transition of hemoglobin, involving a “scissoring and twisting” motion between the two subunits connected via a salt bridge. The presence of this salt bridge is vital for strong interaction with lysosomal enzymes, emphasizing the significance of ionic interactions between the dimer’s subunits in the binding process. Figure 5 represents the 3D image of an animal lectin (galectin) isolated from Homo sapiens. The structures of lectins are obtained from UniProt (The UniProt consortium, 2023). 

### 4.3 Fungal lectins

To date, only 26 different fungal lectins have been characterized based on their structure, indicating a significant gap in our understanding of these bioactive proteins. Approximately 100 crystal structures have been identified, of which 40% are in complex with carbohydrate ligands. These lectins represent 8.5% of all lectins and have been found to possess ten distinct folds, including classical lectin or carbohydrate-binding module (CBM) folds. These folds are prevalent across all living organisms, such as β-trefoil, β-sandwich (including galectin, Ig, and L-lectin types), and the LysM domain.

The cyanovirin-type fold is found in cyanobacteria, while the 6-blade β-propeller is present in soil bacteria, suggesting a potential transfer of genetic material between fungi and soil-dwelling organisms. The calcium-containing β-sandwich of flocculin is exclusively found in yeasts. The integrin-like 7-blade β-propeller is a distinctive feature of lectins in fungi, along with the actinoporin-like fold, also known as the fungal fruit body lectin fold. Descriptions are provided for representatives of the main classes below.

Galectins are a conserved group of β-galactoside-binding proteins found in vertebrates, invertebrates, and certain fungi. The galectins from *Agrocybe aegerita* (AAL) and *Coprinopsis cinerea* (CGL2) exhibit the typical galectin fold, characterized by a β-sandwich structure formed by two parallel, six-stranded, antiparallel β-sheets. One of these sheets creates a concave surface specialized for binding β-galactosides. Although lectins are typically dimeric, the galectins from *C. cinerea* stand out as tetrameric structures, resembling a four-leaf clover assembly (Feng et al., 2010; Butschi et al., 2010; Wälti et al., 2008).

Lectins derived from the fruiting bodies of various fungi, such as *Boletus edulis* (BEL), *Xerocomus chrysenteron* (XCL), *Agaricus bisporus* (ABL), and *Sclerotium rolfsii* (SRL), exhibit an actinoporin-like structure. This fold comprises a β-sandwich formed by two β sheets containing six and four β strands, respectively, connected by a helix-loop-helix motif. These lectins typically form dimers or tetramers, each monomer possessing two distinct binding sites located on either side of the helix-loop-helix motif. The primary binding site is specific for N-acetyl galactosamine (GalNAc), while the secondary binding site is specific for N-acetyl glucosamine (GlcNAc) (Bovi et al., 2011; Birck et al., 2004; Carrizo et al., 2005; Leonidas et al., 2007).

**FIGURE 6:**
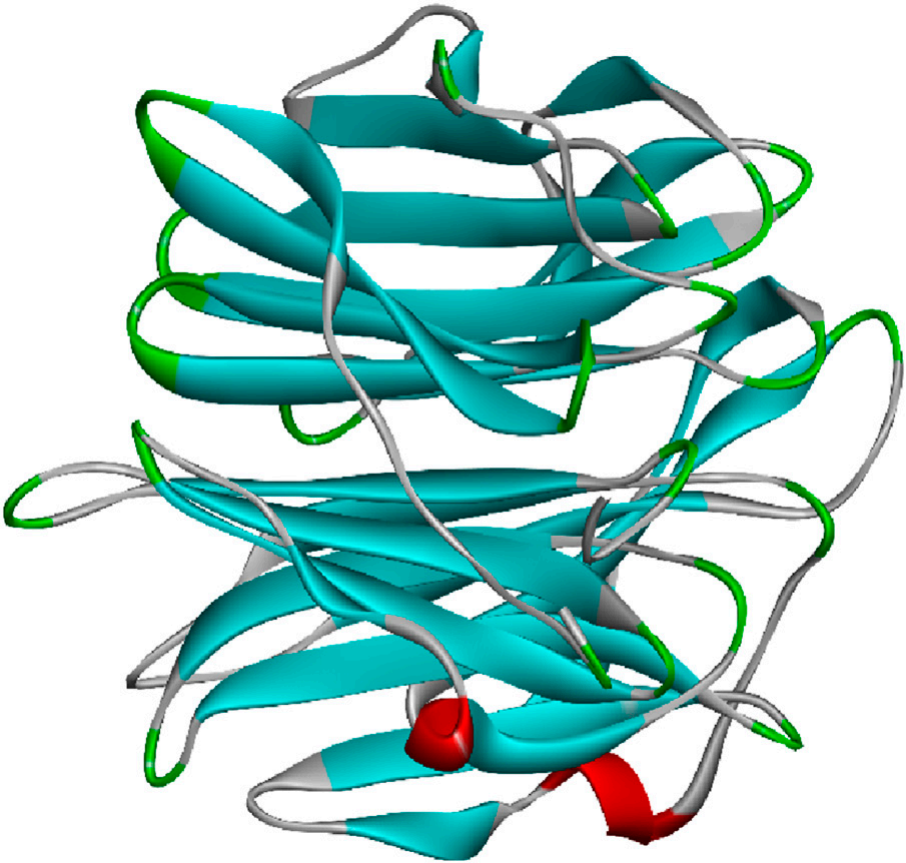
Fungal lectin isolated from *Aleuria aurantia* (orange peel mushroom); structure obtained from UniProt (UniProt ID: P18891); image visualized using Biovia Discovery Studios 2021.

Many fungal lectins share homology with cyanovirin-N homolog (CVNH), a small antiviral lectin produced by the cyanobacterium *Nostoc ellipsosporum*. These lectins are typically monomeric and predominantly fall into type I CVNH, featuring a single copy of cyanovirin-N, like the GzCVNH lectin found in the wheat head blight fungus *Gibberella zeae*. GzCVNH comprises two CVNH repeats, designated as domains A and B, which exhibit a pseudo twofold symmetry. Although both domains can potentially bind carbohydrates, often only one binding site is functional in fungal CVNHs. The structure of *Magnaporthe oryzae* cyanovirin-N homolog lysine motif (MOCNVH-LysM) from *M. oryzae* provides insights into type III CVNHs. In this variant, a LysM domain is inserted between the two CVNH repeats. The LysM domain specifically recognizes chitin oligomers, while the CVNH portion binds oligomannosides in both the A and B domains. Figure 6 represents the 3D image of a fungal lectin isolated from *Aleuria aurantia* (orange peel mushroom). The structures of lectins are obtained from UniProt (The UniProt consortium, 2023).

## 5 Pharmacological applications related to diabetes

### 5.1 Effects on hyperglycemic conditions

The relationship between diabetes mellitus and hyperglycemia is intricate, with hyperglycemia being a hallmark feature of diabetes. In diabetes mellitus, the body either does not produce enough insulin (type 1) or cannot effectively use the insulin it produces (type 2), leading to elevated blood sugar levels (Ramu et al., 2015; Ramu et al., 2016). Hyperglycemia, or high blood sugar, is a common consequence of uncontrolled diabetes and can contribute to a myriad of complications affecting various organs and systems in the body (Maradesha et al., 2022; Ramu et al., 2022). The initial research carried out to assess the potential of lectins to regulate BG levels revealed the efficiency of lectins isolated from plants over other sources. An *in vivo* study found that BG concentration decreased in group III (BG concentration at the end of 4 weeks was 296 ± 89.34); that is, the diabetic group treated with lectin. BG concentration was a little higher than the diabetic group treated with the standard drug glipizide than diabetic control (group II BG concentration of 412.42 ± 111.65 mg/dL at the end of 4th week) at the dosage of 100 mg/kg of *Urtica pilulifera* seed lectin (UPSL) injected intraperitoneally in streptozotocin-induced diabetic rats (Kavalali et al., 2002). Another *in vivo* study carried out in alloxan-induced diabetic female Wistar rats found that ABA at two doses, that is, 150 mg/kg and 200 mg/kg, decreased serum glucose concentration by 15% and 40% at doses of 150 mg/kg and 13% and 43% at 200 mg/kg on the 7th day and 14th day, respectively, compared to the diabetic group (Sawant et al., 2017). The *in vivo* study performed by Rocha et al. (2013) suggested that CrataBL exhibited excellent hypoglycemic activity after 10 days of treatment, with a 56% reduction in BG level at the dosage of 20 mg/kg, whereas the same lectin at the dosage of 10 mg/kg reduced BG level by only 15%. Serum urea levels were decreased by 20.7% and 25.3% at the 10 mg/kg and 20 mg/kg doses, respectively.

According to Gadgoli et al. (2015), serum glucose level was decreased by lectin isolated from *Vigna radiata* bound with zinc (zinc-lectin complex) at a lower dose than individual lectin administered through oral and subcutaneous routes. Another *in vivo* study found that an endophytic fungal lectin isolated from the leaves of *Viscus album* reduced glucose level by 60.76% on the 14th day at a dosage of 400 mg/kg. Kidney function markers like urea and creatinine levels were decreased by 15.9% and 0.08%, respectively, at a 40 mg/kg dosage. Liver function markers such as AST and alanine transaminase (ALT) decreased by 26% and 5%, respectively, at the dosage of 40 mg/kg. After 14 days of treatment, the lectin-treated group showed serum cholesterol and serum triglyceride values of 90.04 ± 0.92 and 98.51 ± 2.02, respectively. The lectin-treated rats showed serum cholesterol and serum triglycerides levels of 90.04 ± 0.92 and 98.51 ± 2.02, respectively, and the lectin-treated diabetic rats showed reduced levels of serum cholesterol (103.54 ± 2.13 mg/dL) and serum triglycerides (124.68 ± 2.49 mg/dL) (Govindappa et al., 2015). Liver glycogen levels were reduced by 21% and 26% after lectin treatments at 150 mg/kg and 200 mg/kg of lectin doses, respectively, whereas the pioglitazone-treated group exhibited glycogen levels reduced by 15% (Sawant et al., 2017).

An *in vivo* study by Grácio et al. (2021) suggested that both native and denatured lectin isolated from the seeds of *Lupinus albus* reduced BG to basal level after 120 min in the lectin-treated groups. Plant lectins are glycoproteins and bind tightly to specific sugar residues and the cell surface. According to Stanely et al. (2000), the mechanism involved in the antihyperglycemic potential of lectin is either to increase pancreatic insulin secretion from β cells or release insulin from bound insulin. The probable mechanism suggested in this research is the direct inhibition of streptozotocin (STZ) by lectin competing with STZ for glucose-associated receptors on the β cell membrane. This *in vivo* work has been designed and carried out systematically using proper methods and controls. However, this study is limited to the antihyperglycemic activity of lectin isolated from seeds and not the other parts of this plant. Hence, more extensive work is required on the antihyperglycemic potential of lectins and other bioactivities like antioxidant, antihypertensive, and anti-obesity effects of lectins isolated from other parts of this plant. Before conducting the *in vivo* study, a toxicity test of the lectin could have been performed. Although there was a decrease in BG concentration, it still did not reach the optimum value; increasing the lectin concentration during the study might have provided more evidence for lectin’s use as an antihyperglycemic agent.

Alloxan monohydrate (AXN) causes type 1 diabetes mellitus. It is toxic to β cells of the pancreas, where it accumulates as glucose analogs (Ighodaro et al., 2017). Furthermore, due to the enhancement of calcium in the cytosol, AXN destroys β cells of the pancreas. Hence, after treatment with lectin, its effect on the β cells of the pancreas and regulation of calcium could have been incorporated into this study with the support of histopathology. As per the acute toxicity test, no mortality was observed, even at 2,000 mg/kg. Although lectin decreased BG level equivalent to that of the standard drug, the reduction percentage was low compared to the standard drug pioglitazone. Performing dose-dependent activity using variable concentrations between 0.0 and 2,000 mg/kg might have allowed the determination of the lowest concentration at which the biological activity could occur for better results and demonstrating this lectin as a potent antihyperglycemic drug. Although the glycogen reduction percentage was less than that of the diabetic group and a little higher than the standard drug pioglitazone, glucose released from glycogen will increase the BG concentration. Hence, the effect of lectin on glycogen metabolic enzymes could have been incorporated. As mentioned in the study, AXN-injected rats exhibited polyuria along with the other symptoms of type 1 diabetes, but the effect of lectin on polyuria, that is, the effect of lectin on urine glucose level, was not incorporated in the study. Blood urea nitrogen (BUN) is one of the kidney damage biomarkers that is associated with the increased risk of incident DM. A two-stage residual inclusion analysis exhibited that, irrespective of Estimated Glomerular Filtration Rate and not epidermal growth factor (eGFR), every 10 mg/dL increase in BUN concentration increased the risk of incident diabetes (Xie et al., 2018). Pham et al. (2012) studied a group of 4,680 patients who were part of a cardiovascular health study without DM and reported that less eGFR is associated with increased insulin resistance. Hence, studying the effect of lectin on BUN is suggested.

The study by Gadgoli et al. (2015) emphasized a Zn-lectin complex and antihyperglycemic activity at lower doses, and a similar study might emphasize the potential of lectins (without zinc) and antihyperglycemic activity by altering the route of administration. Though the results related to the characterization of lectins are mentioned, pictorial and graphical representations of the data were not included in the results and discussion. The antihyperglycemic potential of the zinc lectins complex could be further explored by the STZ model. The insulin-mimicking activity of the lectins using *in vitro in vivo* models to establish mechanisms for antihyperglycemic activity and cholesterol-lowering effect could have been used.

The study by Govindappa et al. (2015), described as an *in vivo* study, was divided into five groups initially, but only four groups were mentioned. Hence, there is a discrepancy in the data. Though five different endophytic fungal species were identified from stem, leaves, and fruit, the study was limited to *Alternaria* isolated from leaves. *Alternaria* from stems and fruits could have been used to further examine the suggested anti-diabetic potential. In the study performed by Grácio et al. (2021), researchers initiated the *in vivo* antihyperglycemic study without an oral acute toxicity test of the lectin; it is suggested to perform a toxicity test before an *in vivo* study to show that the lectin is toxic or non-toxic to the animals. Another *in vivo* study conducted using Wistar rats using plant lectin isolated from the seeds of *V. radiata* plant used subcutaneous and oral administration of lectins at three different doses, 150 mg/kg, 250 mg/kg, and 300 mg/kg. Glibenclamide decreased the BG level and further induced hypoglycemia (approx.79 mg/dL), whereas the lectins (300 mg/kg) and lectin complexed with zinc (150 mg/kg and 250 mg/kg) decreased the BG level along with maintaining the basal level, not leading to hypoglycemia (Gadgoli et al., 2015).

An *in vivo* study by Wang et al. (2012) found that when injected into mice, ABL reached glucose concentration ≤300 mg/dL by the end of the 120 min testing period after 7 days of treatment. Significant differences in the results of glucose tolerance tests among the animal groups were observed on the 14th day by the end of the 120-min testing period. According to the hypothesis of Gutierrez et al. (2011), high glucose tolerance in animals is due to the high peripheral utilization of glucose by anti-diabetic agents, as lectin is a carbohydrate-binding protein. The same principle that applies to plant extracts might also apply to lectin. Although necessary controls were used in the oral glucose tolerance test, the mechanism of glucose tolerance, which provides authentic proof to the study, was not deduced. Insulin levels could have been monitored before and after the glucose tolerance test. In the present study, an oral glucose tolerance test was performed; performing glucose tolerance tests through various modes of administration, such as intra-peritoneal, would have led to a comparative study of the types of glucose tolerance tests. In the *in vivo* study by Wang et al. (2012), an intravenous glucose tolerance test was performed for 120 min, but the researchers could have performed a glucose tolerance test for more than 120 min through various modes of administration. In addition, insulin levels could have been measured after the administration of the drug.

### 5.2 Effects on carbohydrate-digesting enzymes

The most important symptom of DM is hyperglycemia, that is, elevation of BG level. One of the mechanisms is the digestion of carbohydrates by enzymes. Hence, it is necessary to inhibit these enzymes to decrease the BG level. The important enzymes that affect BG levels are alpha-glucosidase, alpha-amylase, sucrase, maltase, lactase, isomaltase, and trehalase (Patil et al., 2022b).

An *in vitro* study by Virounbounyapat (2012) suggested that lectin isolated from *Archidendron jiringa* seeds exhibited a strong α-glucosidase inhibition at three different concentrations of 0 mg/dL, 0.05 mg/dL, and 0.075 mg/dL. The IC_50_ value of 0.031 ± 0.02 mg/dL with an inhibition constant (K_i_) of 1.887 μg/mL was determined. The mechanism of inhibition states that both K_m_ and V_max_ decreased with an increase in the concentration of the lectin hence, lectin inhibited alpha-glucosidase by non-competitive inhibition. Kinetic studies were analyzed using Lineweaver–Burk (LB) and Eaddie–Hofstee (EH) plots.

Another *in vitro* study explained that an endophytic fungal lectin isolated from *Alternaria* species of *Viscum album* inhibited three carbohydrate-digesting enzymes: α-amylase, α-glucosidase, and sucrase with volume of the sample being 10.50 µL, with an inhibition percentage of 85.26%, 93.41% ± 1.27%, and 81.61% ± 1.05% (Govindappa et al., 2015). Another *in vitro* study found that lectin isolated from vent seeds of *Sterculia monosperma* at three different concentrations, 0 mg/dL, 0.05 mg/dL, and 0.075 mg/dL, inhibited α-glucosidase with the reductions in K_m_ and V_max_ being linear with the increasing concentration of lectins. The inhibition constant (K_i_) was 1.39 μg/mL, and it exhibited a non-competitive inhibition (Karnchanatat and Sangvanich, 2014). An *in vitro* kinetic study conducted by Virounbounyapat (2012) analyzed the LB plot and EH plot, but the article only represented the LB plot and not the EH plot. Including the EH plot could support a better understanding of the kinetic studies. The highest α-glucosidase inhibition was by a water-soluble lectin isolated from seeds of *Moringa oleifera* (WSMol) with 93.41% ± 1.27%. Although various anti-diabetic drugs like acarbose, glibenclamide, miglitol, etc., are mentioned in the *in vitro* study by Govindappa et al. (2015), who used untreated enzyme solution as their control, IC_50_ and K_i_ values are not determined, and hence, the mechanism of inhibition cannot be determined. Meanwhile, Virounbounyapat (2012), and Karnchanatat and Sangvanich (2014) mentioned the mechanism of inhibition but did not mention controls. Moreover, the researchers focused on α-glucosidase more than the other enzymes. As insulin is the key hormone in the regulation of BG level, it is important to study the relationship between insulin level and the inhibitory mode of enzyme(s). Future studies could focus on how insulin secretion is affected after the inhibition study. *In silico* studies could explain the inhibition pattern, active site binding, and residue-specific binding in detail. Including *in silico* studies of the lectin–enzyme interactions would support *in vitro* inhibition studies.

### 5.3 Effects on oxidative stress

Diabetes, especially type 2, is linked to increased oxidative stress due to high glucose levels in the bloodstream. This leads to the overproduction of reactive oxygen species (ROS), which can damage cellular components. Oxidative stress can also contribute to the development and progression of diabetes. Therefore, it is crucial to target oxidative stress pathways in diabetes management and prevention (Ramu et al., 2022). An *in vitro* and *in vivo* study by Alves et al. (2020) suggested that three different doses of BSL, 150 μg/kg, 300 μg/kg, and 600 μg/kg, in male Wistar rats activated antioxidant enzymes: superoxide dismutase, catalase, and glutathione peroxidase. Blood analysis from 30 days, 60 days, 90 days, and 120 days suggested that in the BSL-treated groups at all doses, a significant increase in enzyme activity was observed compared to the negative control and metformin groups. No significant changes were observed in the catalase enzyme activity in all the groups. An insignificant increase in glutathione peroxidase enzyme activity was observed across all BSL-treated groups compared to the negative and positive controls in the blood collected from the posterior limbs at 30 days, 60 days, 90 days, and 120 days. However, after 90 days and 120 days, an insignificant difference in the 600 μg/kg BSL group compared to other BSL groups (150 μg/kg and 300 μg/kg) was observed.

Oxidative stress may also initiate the generation of ROS along with other proinflammatory cytokines and chemokines in the β cells, disrupting the blood flow into them and destroying their functions (Dedon and Tannenbaum, 2004; Ehses et al., 2007; Akash et al., 2012). Lectin showed probable and promising results concerning superoxide dismutase and glutathione peroxidase whereas it did not affect catalase. Examining lectin-activated antioxidant enzymes *in vitro* and constructing *in silico* models using bio-computational tools like molecular docking and network pharmacology could provide concrete proof and an excellent future perspective. This study only focused on lectin and its effect on antioxidant enzymes; there are many other targets related to oxidative stress, proinflammatory pathways, etc. Hence, it is suggested that lectins and their interaction with ROS generation and proinflammatory cytokines be studied. Oxidative stress leads to the initiation of microvascular and cardiovascular diabetes complications. Aberrations in the metabolism of diabetes lead to the excess production of mitochondrial superoxide, which is the foremost intermediary of tissue damage in diabetes, leading to the stimulation of five pathways involved in the complications of pathogenesis and deactivation of two anti-atherosclerotic enzymes, endothelial nitric oxide synthase (eNOS) and prostacyclin synthase. By these pathways, intracellular ROS increases and causes defective angiogenesis in response to ischemia, leading to activation of several proinflammatory pathways and causing lifelong epigenetic changes leading to persistent expression of proinflammatory genes even though glycemia is normalized (“hyperglycemic memory”) (Giacco and Brownlee, 2010). Hence, lectins and interaction with five pathways, two anti-atherosclerotic enzymes, and proinflammatory pathways could be studied using bio-computational approaches such as molecular docking and system biology, which could support the *in vitro* and *in vivo* studies. With these studies, a new therapy for diabetes-mediated atherosclerosis and diabetes-linked inflammation could be designed with lectins.

### 5.4 Effects on insulin synthesis

T1DM is an autoimmune disorder that is due to the selective, specific destruction of β cells, which produces insulin, without pathological changes of other Langerhans cells of the pancreas (Atkinson et al., 2014). Macrophages also critically mediate islet inflammation due to their capability to secrete cytokines, such as interleukin 1 β (IL-1β) and tumor necrosis factor-alpha (TNF-alpha), and produce ROS (Hutchings et al., 1990; Willcox et al., 2009). Maedler et al. (2003) proposed an ALPHA pathway that suggested that hyperglycemia may induce the production of interleukin-1β (IL-1β) by stimulating pro-apoptotic receptor-free fatty acids (FFAs) on β-cells. IL-1β, after secreting initially, regulates its production in pancreatic β-cells by auto stimulation. However, this process also increases nitric oxide production, which leads to a reduction in adenosine triphosphate (ATP) concentration in the mitochondria, further leading to β-cell dysfunction and reduced insulin secretion (Arafat et al., 2007; Böni-Schnetzler et al., 2008; Böni-Schnetzler et al., 2009).

According to Kavalali et al. (2002), UPSL prevented cellular damage to the pancreas when injected intraperitoneally at a dose of 100 mg/kg. In diabetic groups (group II, III, and IV), the numbers of islet and islet cells were found to be lower than normal (group I); only the average diabetic control group (group II) was found to be statistically significant (p < 0.05). In the diabetic glipizide group (group IV), the size of the islets was found to be significantly smaller than in the other groups. Another *in vivo* study found that lectin isolated from the fungus *Agaricus bisporous* at 10 mg/kg increased β-cell proliferation replication via regulation of cell cycle proteins and upregulation of genes involved in β-cell proliferation. The pancreas section stained for bromodeoxyuridine (BrdU) and insulin showed a remarkable increase in the number of BrdU-positive cells after treatment by *A. bisporous* lectin (ABL). A significant 2.7-fold increase in β cell proliferation was observed. In ABL-treated mice, small islets had greater proliferation rates than the larger ones. ABL-treated mice had elevated serum insulin concentrations compared to PPx control mice. Absolute β-cell mass was significantly elevated by ABL treatment. Remarkable increases in cyclin-dependent kinase (cDK4) messenger ribonucleic acid (mRNA) and protein concentrations were observed over PPx control using real-time polymerase chain reaction (RT-PCR). mRNA and protein concentrations of cyclin D1 and cyclin D2 were also increased. Cyclin D2 was upregulated in ABL-treated mice. In addition, in ABL-treated mice, phosphorylation of rGST-Rb was significantly higher in islet lysates than in the PPx control mice, indicating the activation of Cdk4. Significant enhancements in insulin, GLUT-2, and glucokinase mRNA levels after ABL treatment was observed (Wang et al., 2012). In an *in vivo* study by Alves et al. (2020), BSL decreased insulin resistance when administered orally at three different doses, that is, 150 μg/kg, 300 μg/kg, and 600 μg/kg. The homeostatic model assessment (HOMA) method was used to evaluate insulin resistance; on day zero, all groups were within the same value of insulin resistance, with 5% significance. At 30 days, 60 days, 90 days, and 120 days, a significant decrease in IR in the BSL and metformin-treated groups compared to the negative control was noted. The HOMA-β method was used to evaluate the functional capacity of β-cells, suggesting that at the beginning, all groups experienced insulin hypersecretion. However, with BSL treatment at three different doses, there was a decrease in the normal group (increased hypersecretion), and the metformin group remained constant. An *in vivo* study by Vera-Nuñez et al. (2021) suggested that a water-soluble lectin isolated from seeds of *Moringa oleifera* (WSMol) treatment was sufficient to decrease the insulin resistance in the T2DM group as well as control non-diabetic group after 3 weeks of WSMol treatment at the dosage of 5 mg/kg.

Although Wang et al. (2012) did extensive work on lectins and β-cell proliferation, a few drawbacks can be observed. They did not mention the mode of administration of lectins for their *in vivo* study, and toxicity tests could have been performed before the actual study. They could have studied the β-cell proliferation at different lectin concentrations. β-cells have human glucose transporter (GLUT 1 and 2), mice GLUT 2 and hexokinase-glucokinase (Heimberg et al., 1995; Ahrén, 2000; McCulloch et al., 2011; Doliba et al., 2012; Bonner et al., 2015; Thorens, 2015). Studying glucose uptake by GLUT receptors, levels of hexokinase-glucokinase, and the lectin–enzyme relationship through *in silico* models would provide more information. In an *in vivo* study by Kavalali et al. (2002), researchers calculated the number of islets, islet size, cells per islet, and islet diameter, but they did not explain the effect of lectin treatment on islets of β-cells; hence a more detailed study is required concerning lectin and proliferation of β cells, monitoring of insulin levels after lectin treatment is suggested. In the *in vivo* study by Alves et al. (2020), researchers used the HOMA-β method to evaluate the capacity of β-cells, but they did not study the capacity through a molecular biological approach like the study of β cell proliferation (with cell cycle). How it binds to insulin receptors could have been studied using *in silico* models. In the *in vivo* study by Vera-Nuñez et al. (2021), WSMol treatment was sufficient in decreasing insulin resistance. Researchers have performed an intra-peritoneal insulin tolerance test (IPITT), but the insulin level was not evaluated during the study. Structural analysis of the pancreas after the lectin treatment, especially the β-cells, could have been conducted using a histopathological approach.

### 5.5 Effects on body weight and nutrition

The relationship between diabetes mellitus and body weight and nutrition is intricate and pivotal. Excessive body weight, especially abdominal adiposity, significantly increases the risk of developing type 2 diabetes mellitus (Usha et al., 2023). Conversely, adopting a balanced diet rich in fiber, whole grains, fruits, and vegetables can aid in weight management and glycemic control and play a crucial role in diabetes prevention and management (Ramu et al., 2022). An *in vivo* study conducted in male Wistar rats showed that when injected intraperitoneally at the dosage of 100 mg/kg, UPSL increased the body weight from 130 ± 18.26 g on an initial day to 193 ± 55.28 g after 4 weeks. Food intake and fluid intake amounts were higher in diabetic groups than normal (Kavalali et al., 2002). In another *in vivo* study by Govindappa et al. (2015), a lectin isolated from endophytic leaf fungi increased the body weight of male albino Wistar rats by 8.5%, which is significant, when administered orally at a 400 mg/kg dosage. According to (Sawant et al., 2017). ABA increased food intake in female Wistar rats by 103% and 162% at doses of 150 mg/kg and 200 mg/kg, respectively. The same lectin increased the body weight of the female albino Wistar rats at 150 mg/kg by 17% and 31% on the 7th day and 14th day, respectively; at 200 mg/kg, the rats’ body weight increased by 20% and 32% on the 7th day and 14th day, respectively. ABA at a 200 mg/kg dosage exhibited a significant increase in body weight.

Researchers have observed the effect of lectins on body weight, food, and fluid intake by administering the lectins intra-peritoneally and orally; the effects of lectins on body weight, food, and fluid intake through other modes of administration, such as subcutaneous, intravenous, etc., remain to be determined. Research links less water intake to an increased diagnosis of hyperglycemia, and the probable mechanism is related to the hormonal intervention of renin-angiotensin-aldosterone system (RAAS), anti-diuretic hormone (ADH), and cortisol and pituitary hormone (Johnson et al., 2017). Hence, lectin and hormone interaction could be studied using both *in vivo* and *in silico* approaches. Food intake is extensively related to the GI tract hormone ghrelin (Kojima et al., 1999; Tschop et al., 2000; Wren et al., 2000), and the lectin and ghrelin relationship could be a future perspective. Diabetes is associated with weight loss due to the catabolism of fats and proteins; hence, lectin and its relationship with the enzymes involved in lipid metabolism and protein metabolism could be studied by researchers through *in silico* approaches like network pharmacology and system biology. These approaches could provide molecular-level evidence of lipid and protein metabolism.

### 5.6 Effects on cholesterol, glycogen, and other biomarkers

The relationship between diabetes mellitus, cholesterol, and glycogen is intricate and multifaceted. Diabetes mellitus, particularly type 2, often accompanies dyslipidemia, characterized by elevated levels of cholesterol and triglycerides, which contribute to cardiovascular complications (Firdose et al., 2023). Additionally, an impaired insulin function in diabetes disrupts glycogen synthesis and storage, leading to abnormal glucose metabolism and further exacerbating the metabolic imbalance. Consequently, managing both cholesterol levels and glycogen storage becomes crucial in mitigating the risks associated with diabetes mellitus (Bodaghi et al., 2023). Lectins exhibited an anti-hypercholesterolemic effect by decreasing the serum cholesterol level by 16% and 33% at 150 mg/kg for 7 days and 14 days dosage and by 19% and 36% at a concentration of 200 mg/kg on the 7th day and 14th day, respectively. Although serum cholesterol levels were not completely normalized, the changes were comparable to standard pioglitazone treatment, and lectin was able to control the effects of high cholesterol (Sawant et al., 2017).

Individual lectin and lectin complexed with zinc reduced the serum cholesterol and triglyceride levels. However, lectin complexed with Zn exhibited higher effects at lower doses than lectins alone. Lectin and lectin complexed with zinc re-established liver glycogen levels (Gadgoli et al., 2015). Denatured γ-conglutin exhibited similar values to the normal control, whereas native γ-conglutin showed a 10% reduced value for ALT. For AST, both the lectins, that is, the denatured and the native, exhibited lower values than normal, which is not significant but much lower than the metformin group (50% and 40%, respectively). Compared to the normal group, all the other groups (excluding the hyperglycemic group) exhibited increased blood insulin levels. In the case of creatinine, the group treated with denatured γ-conglutin, the metformin group, and the hyperglycemic group exhibited similar values but lower than the normal group. Meanwhile, the group treated with native γ-conglutin exhibited the highest creatinine values, with no significant difference compared to the normal group. All the groups in the study exhibited similar urea values. In the case of cholesterol and triglycerides, the normal control group exhibited similar values of cholesterol compared to that of the groups treated with native γ-conglutin, whereas groups treated with denatured lectin exhibited a slightly lower value. The metformin and hyperglycemic groups exhibited similar total cholesterol values. Both lectin-treated groups showed increased high-density lipoprotein (HDL) values. Concerning TG, no difference between normal, hyperglycemic, and denatured lectin-treated groups (230 mg/dL) was noted. The group treated with native lectin showed a higher value (290 mg/dL) than the normal group. The metformin group exhibited lower TG levels (200 mg/dL) (Grácio et al., 2021).

The results suggest that native γ-conglutin may not be effective in reducing the creatinine level. As γ-conglutin did not affect the urea level, it is suggested that the effect of lectin on the urea level could examined in greater detail.

### 5.7 Effects on gut microbiome and diabetes mellitus

Although lectins are not directly linked to DM, they are indirectly connected through their influence on gut microbiota, which is associated with several other health issues. For instance, the C-type lectins play a crucial role in innate immunity by regulating the growth of enteric pathogens. They are expressed by various small intestinal epithelial lineages. Two such lectins, REG3γ and REG3β, protect against infection by specific bacterial pathogens like *Enterococcus faecalis*, *Yersinia pseudotuberculosis*, and *Listeria monocytogenes* (Cheng et al., 2019). Though not directly involved, these pathogens can occur in multiple myeloma patients and are responsible for secondary diabetes (Hu et al., 2013). Therefore, lectins could be considered as potential medications for gut health. Additionally, evidence suggests that C-type lectins also play a role in defending against syncytium endosymbionts by preventing pathogen colonization (Wang et al., 2020). Studies on Vil-Myd88Tg mice revealed that both MyD88 pathway activation and TLR recognition of syncytium endosymbionts are necessary for C-type lectin secretion (Ahmed et al., 2021; Cheng et al., 2019). Moreover, research using a pipeline method demonstrated that the removal of dietary microbiota-accessible carbohydrates (MACs) led to a reduction in mucus thickness in the distal colon, increasing microbial proximity to the epithelium and enhancing REG3β expression, which is an inflammatory marker. This aligns with earlier findings showing that MyD88 is crucial for syncytium endosymbiont-induced expression of antimicrobial genes in colonic epithelial cells (Cheng et al., 2019). MyD88 is a pathway that plays a role in diabetes-related inflammation in the heart and retina. In a rat model of type 2 diabetes mellitus, TLR4/MyD88/NF-κB signaling led to the production of tumor necrosis factor-α, interleukin-6, and monocyte chemoattractant protein-1, which caused heart and liver complications (Tian et al., 2021). In addition, hyperglycemia activates TLRs-MyD88 in cardiomyocytes and heart tissues, which can cause cardiomyocyte hypertrophy and fibrosis. However, pharmacologic inhibition of MyD88 can have anti-inflammatory, anti-hypertrophic, and anti-fibrotic effects in the hearts of diabetic mice (Luo et al., 2022; Tang et al., 2013). This indicates that MyD88 may be a potential therapeutic target for diabetic cardiomyopathy.

In addition to bacteria, the mammalian gut harbors a diverse fungal community that interacts with the immune system through receptors like Dectin-1. Research on mice lacking Dectin-1 revealed an increased susceptibility to colitis due to altered responses to indigenous fungi. Similarly, a gene polymorphism for Dectin-1 (CLEC7A) was found to be strongly associated with severe ulcerative colitis in humans (Iliev et al., 2012; Wang et al., 2022). Dectin-1 (CLEC7A) is a small protein that is important for cell activation and inflammatory cytokines in high-concentration glucose and palmitate acid (HG + PA)-challenged macrophages. Studies show that Dectin-1 mediates diabetes-induced cardiomyopathy by regulating inflammation. For example, Dectin-1 deficiency in macrophages has been associated with an anti-inflammatory phenotype and improved insulin sensitivity in adipocytes.

Interestingly, recent insights reveal that lectins also offer numerous benefits for gut health and metabolism in animals, sparking interest in exploring their potential medical applications other than DM (Santos et al., 2021). Dietary lectins can survive the gut and affect the intestine’s bacterial flora, hormone secretion, and overall health. They bind to specific receptors on gut cells, cause chemical reactions, and trigger immune responses. Animal studies suggest a promising opportunity to apply this knowledge to clinical practice (Pusztai, 1993; Freed, 1999).

Many human lectins found on mucosal surfaces could bind to microbes, although the exact microbial targets of these lectins are not well-understood. The interaction between human hosts and their resident microbes primarily occurs through glycans on bacterial cell surfaces (Kumari et al., 2022; Huligere et al., 2023). Human soluble carbohydrate-binding proteins (lectins) recognize these glycans to identify specific microbial strains (McPherson et al., 2023). However, the precise targets of lectins among commensal microbial species are poorly understood, and how lectins bind within microbial communities remains unexplored.

**FIGURE 7:**
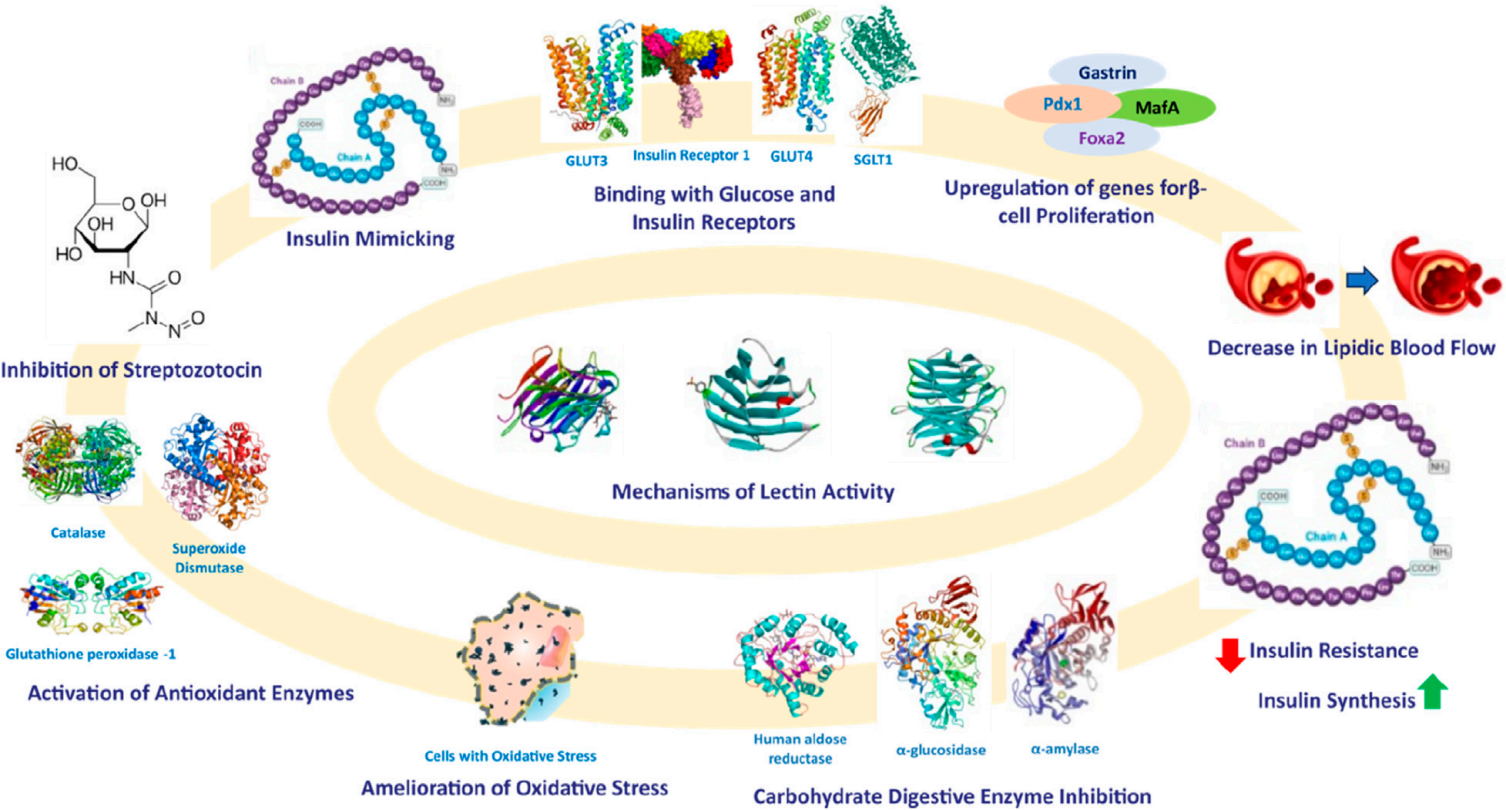
Mechanisms of antihyperglycemic activities of lectins.

Based on these studies, it can be inferred that lectins could be utilized to identify gut microbiota by recognizing specific glycans present in gut microbes. Additionally, research has established that the gut microbiome significantly impacts the development of diabetes mellitus (Zhang et al., 2021; Li et al., 2020). Therefore, lectins sourced from various origins can be isolated, purified, and employed as a diagnostic tool to identify pathogens in diabetic patients by interacting with glycans. After identifying the gut microbes involved in triggering oxidative stress and a proinflammatory response, the gut microbiome can be normalized through dietary interventions, such as the consumption of prebiotics and probiotics. The mechanisms of reported lectins with their anti-diabetic effects and mechanisms are shown in Figure 7.

## 6 Toxicity analysis

Of the 11 research articles examined, only 3 (27%) conducted toxicity tests prior to the *in vivo* studies. The remaining eight articles (73%) did not include toxicity tests. It is highly recommended that researchers perform toxicity tests to determine whether lectin is toxic or non-toxic to animals. One *in vivo* study performed toxicity tests by creating four groups with five animals in each group. A toxicity test was performed by treating a maximum dose of up to 1,200 mg/kg orally. The initial dosage started from 400 mg/kg. Starting the toxicity test from a lesser dosage to the highest dosage, that is, from 100 mg/kg, might provide more information that could be interpreted and understood easily as suggested. Researchers could have mentioned the control group in their toxicity study (Govindappa et al., 2015). In the *in vivo study* performed by Gadgoli et al. (2015), the toxicity test was done directly by injecting a higher dosage of 2,000 mg/kg crude lectins and lectin complexed with zinc without starting from a lower dosage. Hence, it is suggested that initializing toxicity tests with a lesser dosage would provide linear information. In another study, the toxicity test was performed using three animals (Sawant et al., 2017). Researchers initiated the test with 175 mg/kg, which is appreciated, and rapidly increased the dosage to 550 mg/kg and then to a very high dosage of 2,000 mg/kg without studying the toxicity at intermediate doses. Hence, it is suggested to perform toxicity testing at all doses within the range considering the intermediate doses.

## 7 Meta-analysis

Six *in vivo* studies with an appropriate mention of sample size were considered for the meta-analysis (Kavalali et al., 2002; Govindappa et al., 2015; Sawant et al., 2017; Alves et al., 2020; Vera-Nuñez et al., 2021; Rocha et al., 2013). The sample size is one of the important parameters for meta-analysis. Hence, researchers could focus on sample size during their research.

The analysis based on six studies is shown in the funnel plot. The studies in the analysis are assumed to be a random sample from a universe of potential studies, and this analysis will be used to make an inference about that universe. The random-effects model was employed for the analysis. The mean effect size is 0.338, with a 95% confidence interval of 0.299–0.378.

The Q-statistic tests the null hypothesis that all studies in the analysis share a common effect size. If all studies shared the same true effect size, the expected value of Q would equal the degrees of freedom. The Q-value is 0.006 with five degrees of freedom. Because the Q-value is less than the degrees of freedom, the amount of between-study variance in the observed effects is less than we would expect to see based on sampling error alone. Therefore, the variance of true effects is estimated as zero, and all heterogeneity indices (I-squared, tau-squared, and tau) are set to zero. The I-squared statistic is 0%, which tells us that some 0% of the variance in observed effects reflects variance in true effects rather than sampling error.

Publication bias: The *p*-values for the meta-analyses for six studies are less than 0.05, which indicates a significant publication bias in the study subject; that is, Egger’s test, *p* < 0. 0001.

**FIGURE 8:**
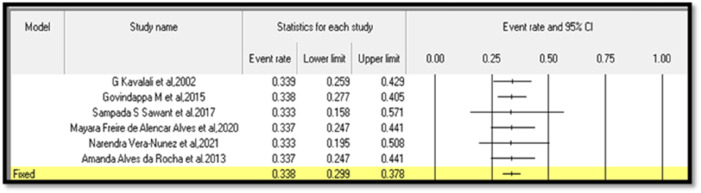
Forest plot based on six studies.

**FIGURE 9:**
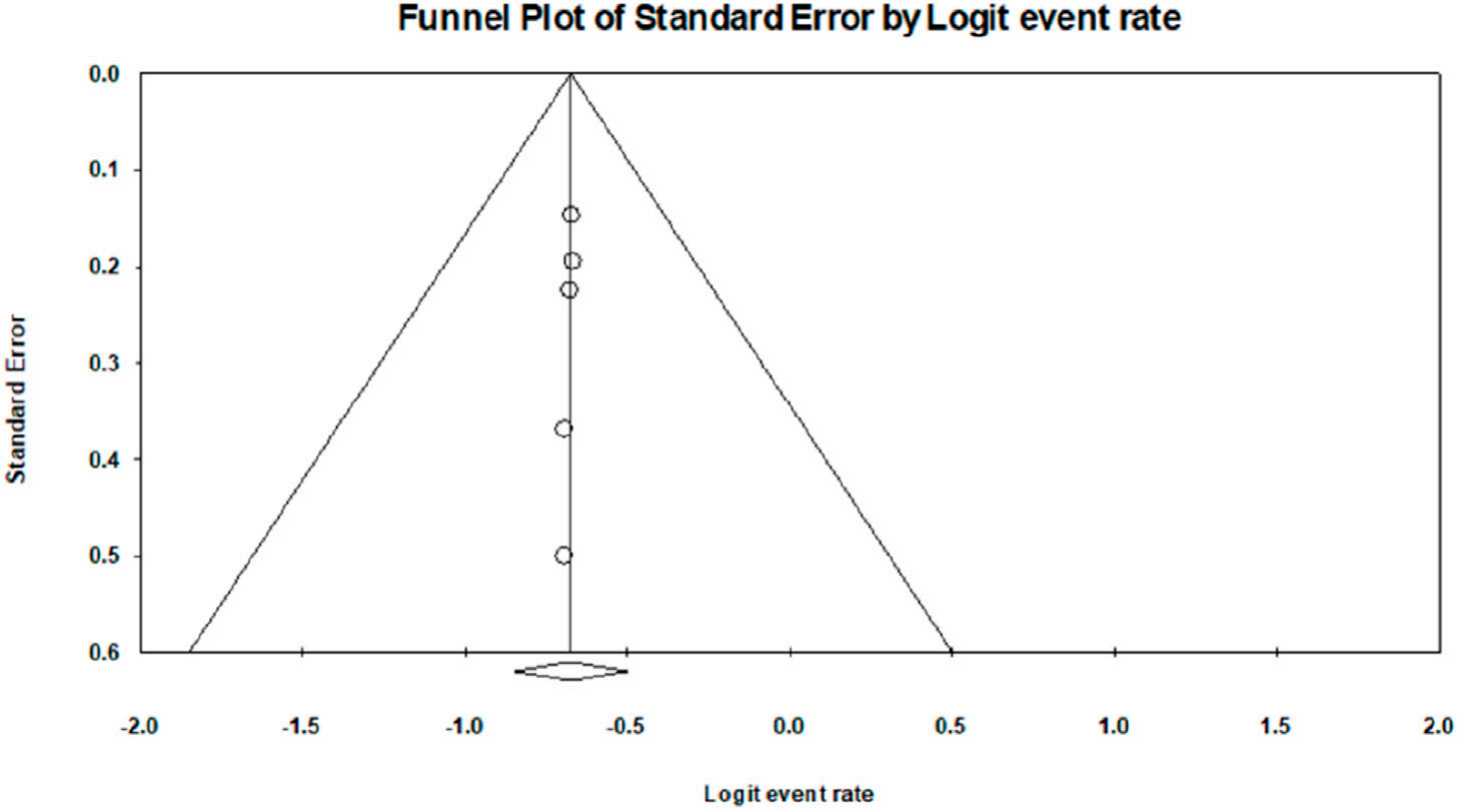
Funnel plot for publication bias.

Continent-wise analysis related to lectins and their anti-diabetic properties explained that 55% of the research work related to lectins and anti-diabetic properties was carried out in Asia, 18% of the research work related to anti-diabetic properties of lectins was carried out in Europe, and 27% of the research work related to lectins and anti-diabetic property was carried out in South America. Because Asia has rich flora and fauna with various medicinal properties, most of the research work related to lectins and anti-diabetic properties is carried out on the largest continent. In contrast, 45% of the research work on lectins and anti-diabetic properties has been carried out in Europe and South America in combination, which could be due to less flora and fauna. The forest plot based on six studies is shown in Figure 8, and the funnel plot for publication bias is shown in Figure 9.

## 8 Future perspectives

Based on the information obtained during the data extraction and reviewing process, the authors noted a few crucial perspectives in every stage of the research methodologies. Although studies have used plants as a source of lectins, the authors suggest that all the parts of the plants should be used separately for the extraction, viz., leaves, stem, root, flower, seeds, etc. In addition, different extracts should be sent for chromatography analyses to detect the maximum number of lectins. These analyses could reveal the maximal solubility of lectins, and the same extract can be used to isolate a particular lectin. After the extraction, both the extract and the isolated lectin should be subjected to toxicity analysis using different concentrations. The toxicity analysis should be done using suitable cell lines. None of the reviewed studies focused on the structural elucidation of lectins, so a primary focus should be on the structural elucidation of lectins, which could be done using nuclear magnetic resonance (NMR) and Fourier transform infrared (FTIR) spectroscopy analyses.

The articles reported herein lack *in silico* studies, as they provide preliminary solid proof of the research work. The insulin-mimicking activity of lectins could be proven through *in silico* models. Targets like GLUT receptors, enzymes and their interaction with lectins could be examined using molecular docking and structure-activity relationship (SAR) studies, which would provide solid scientific evidence. In addition to the *in silico* interactive studies like molecular docking, compound stability could be studied using molecular dynamics simulation, binding free energy calculations, principal component analysis (PCA), and free energy landscape (FEL) analysis. These *in silico* analyses would provide the exact binding-specific stability of the lectins inside the protein targets. The authors suggest using bio-computational approaches like network pharmacology and system biology to understand the protein and lipid metabolism of the cell/animal models upon treatment with lectins. Approaches like qPCR and metagenomics could be used to analyze the gene expression in cell/animal models upon the treatment with lectins. These studies would depict the pharmacological effect of lectins at the genomic and translational levels.

As DM is associated with multiple complications like hypertension, oxidative stress, altered lipid profile, obesity, etc., advanced studies targeting antihypertensive, antioxidant, anti-hyper-lipidemic, and anti-obesity properties of lectins need to be performed. As DM is a multifactorial disorder, its treatment should also be a multifactorial approach. Multiple parameters like insulin, blood glucose, total cholesterol (TC), triglycerides (TG), low-density lipoproteins (LDL), very-low-density lipoproteins (VLDL), high-density lipoproteins (HDL), and liver biomarkers like ALT and AST could be included. Cell-free reactive oxygen species (ROS) depletion analysis using DPPH (2,2-diphenyl-1-picrylhydrazyl) and ABTS (2,2′-azino-bis-(3-ethylbenzothiazoline-6-sulfonic) acid) could also be done. In addition, cell culture-based nitroblue tetrazolium (NBT) can also be done to evaluate the antioxidant potential of the lectins.

Advanced studies are required to evaluate the effect of lectins on glucose tolerance and glucose uptake. Most studies mentioned in this review are limited to plant lectins and, more specifically, to seed lectins and their anti-diabetic properties. Hence, a more detailed study is required to identify potent sources of lectins in plants other than seeds and other sources such as fungi, algae, and animals. Future studies could focus on lectins and insulin resistance as well as lectins and autoimmunity, as type 1 diabetes mellitus (T1DM) is due to autoimmune reactions. The current understanding of the effect of lectins is limited to three enzymes of glucose metabolism; researchers could study its effect on other glucose metabolic enzymes that are involved in glucose homeostasis. Studies can also focus on the effect of lectins on oxidative stress, as it is one of the causes of DM. Diabetes is also associated with hypertension, wherein angiotensin-converting enzyme (ACE-2) plays an important role related to hypertension. Hence, the effect of lectins on ACE-2 could be evaluated with the expectation of identifying a new therapy for hypertension.

The authors noticed a scarcity of studies focused on the pharmacological effect of lectins on the gut microbiome. Because DM and its prevalence in the human body are associated with the gut, microbiota from the gut region plays a crucial role in diabetes mellitus diagnosis and treatment. Although some progress has been made in inhibiting molecular targets in the gut microbiota with lectins, a direct link to diabetes mellitus has not yet been established. Furthermore, the authors noticed no clinical trials using lectins isolated from different sources. Though *in vivo* studies have proved the antihyperglycemic potential, no work has reported conducting dose-dependent human clinical trials. Therefore, research should focus on this to establish clinical trials using lectins.

## 9 Conclusion

Lectins have become key biomolecules in the field of anti-microbials, agriculture, and chromatography. However, deciphering their anti-diabetic properties has yet to reach its complete potential. Extensive research has been undertaken to evaluate their antihyperglycemic activity and associated complications. However, several aspects are lacking to conclusively state their ability to be used as a potent antihyperglycemic drug. In addition, although literature is available on the anti-diabetic potential of lectins, very few articles have examined the effect of lectins on glucose tolerance, oxidative stress, glucose uptake, and diabetic complications like nephropathy, neuropathy, cardiomyopathy, retinopathy, and wound healing properties. In the present study, we have described the various gaps that need to be bridged to understand the efficacy of lectins in treating DM. Furthermore, we suggest employing *in silico* methods to understand the role and ability of lectins to modulate specific targets/pathways to bring about antihyperglycemic effects. In conclusion, dose-dependent clinical studies should be performed with human cell lines, followed by human trials, which would help to provide a better understanding of the protein and its anti-diabetic effects and possibly create a new therapy for DM.

## Data Availability

The raw data supporting the conclusions of this article will be made available by the authors, without undue reservation.
